# A census of *P*. *longum*’s phytochemicals and their network pharmacological evaluation for identifying novel drug-like molecules against various diseases, with a special focus on neurological disorders

**DOI:** 10.1371/journal.pone.0191006

**Published:** 2018-01-10

**Authors:** Neha Choudhary, Vikram Singh

**Affiliations:** Centre for Computational Biology and Bioinformatics, School of Life Sciences, Central University of Himachal Pradesh, Himachal Pradesh, India; Hokkaido Daigaku, JAPAN

## Abstract

*Piper longum* (*P*. *longum*, also called as long pepper) is one of the common culinary herbs that has been extensively used as a crucial constituent in various indigenous medicines, specifically in traditional Indian medicinal system known as Ayurveda. For exploring the comprehensive effect of its constituents in humans at proteomic and metabolic levels, we have reviewed all of its known phytochemicals and enquired about their regulatory potential against various protein targets by developing high-confidence tripartite networks consisting of phytochemical—protein target—disease association. We have also (i) studied immunomodulatory potency of this herb; (ii) developed subnetwork of human PPI regulated by its phytochemicals and could successfully associate its specific modules playing important role in diseases, and (iii) reported several novel drug targets. P10636 (microtubule-associated protein tau, that is involved in diseases like dementia etc.) was found to be the commonly screened target by about seventy percent of these phytochemicals. We report 20 drug-like phytochemicals in this herb, out of which 7 are found to be the potential regulators of 5 FDA approved drug targets. Multi-targeting capacity of 3 phytochemicals involved in neuroactive ligand receptor interaction pathway was further explored *via* molecular docking experiments. To investigate the molecular mechanism of *P*. *longum*’s action against neurological disorders, we have developed a computational framework that can be easily extended to explore its healing potential against other diseases and can also be applied to scrutinize other indigenous herbs for drug-design studies.

## Introduction

Healing with medicinal plants is an ancient idea. Secondary metabolites of various plants have been traditionally utilized for the betterment of human health. Plants belonging to genus Piper are amongst the most important medicinal plants used in various systems of medicine. More than 1,000 species belong to this genus and *P*. *longum* is one of the most well-known species amongst them, including *Piper nigrum* and *Piper bettle*. *P*. *longum* forms an active constituent of the widely used Ayurvedic poly-herbal formulation “Trikatu” [1]. The widespread use of this herb in different formulations as documented in ancient Ayurvedic manuscripts such as Caraka samhita [2], Susruta samhita [3] Vagbhata’s astanga hrdayam [4] etc. suggests its vital importance in traditional Indian medicinal system.

*P*. *longum* is an indigenously growing plant in India and is also cultivated in the tropical and subtropical regions of Asia and Pacific islands [5]. It is usually cultivated for its fruit which is dried and used as a spice. The plant grows into a shrub with large woody roots, numerous creeping and jointed stems that are thickened at the nodes. Leaves are without stipules and spreading in nature. Fruits are small, oval shaped berries and grow as spikes that are collected after maturation. Dried form of these spikes makes “pippali” while the root radix is known as “pippalimula”. The dietary piperine is known for its bioavailability and digestion enhancing properties. *In vitro* studies have shown the role of piperine in relieving oxidative stress by quenching free radicals and reactive oxygen species. While it is known to act as an anti-mutagenic and anti-tumor agent [6], anti-diarrheic and anti-dysenteric properties of this spice enhance its medicinal value [7]. The pharmacological properties of this plant also include anti-oxidant, anti-inflammatory, hepatoprotective, immunomodulatory, anti-microbial, anti-platelet, anti-hyperlipidemic, analgesic, anti-depressant, anti-amoebic, anti-obesity, radioprotective, cardioprotective and anti-fungal [8], [9], [10], [11]. Methanolic extract of this fruit has been reported to be involved in memory repair and improving memory performance by an *in vitro* model [12]. Clinical studies have revealed the efficacy of this plant in the treatment of bronchial asthma in children [13], [14]. Anti-diabetic activity of the roots has also been reported [15]. It is widely used as an important constituent in various Ayurvedic medicines to cure diseases like leprosy and tuberculosis and is also used in the treatment of cough, dyspnea, cardiac and spleen disorders, chronic-fever, gout, rheumatic pain etc. [16].

In recent years, the advancement in chemistry, pharmacology and systems biology has created a new paradigm for the drug discovery known as network pharmacology [17]. Integration of traditional knowledge of medicines with recent *in silico* approaches has led to the identification of novel natural drug compounds. The approach has recently gathered much attention by the research community as network pharmacology based studies have been widely used to explore the medicinal activities of herbs like *Withania somnifera* [18] and formulae like QiShenYiQi [19], Gegen Qinlian decoction [20] etc. to understand their molecular level effect in the treatment of syndromes or diseases.

**Fig 1:**
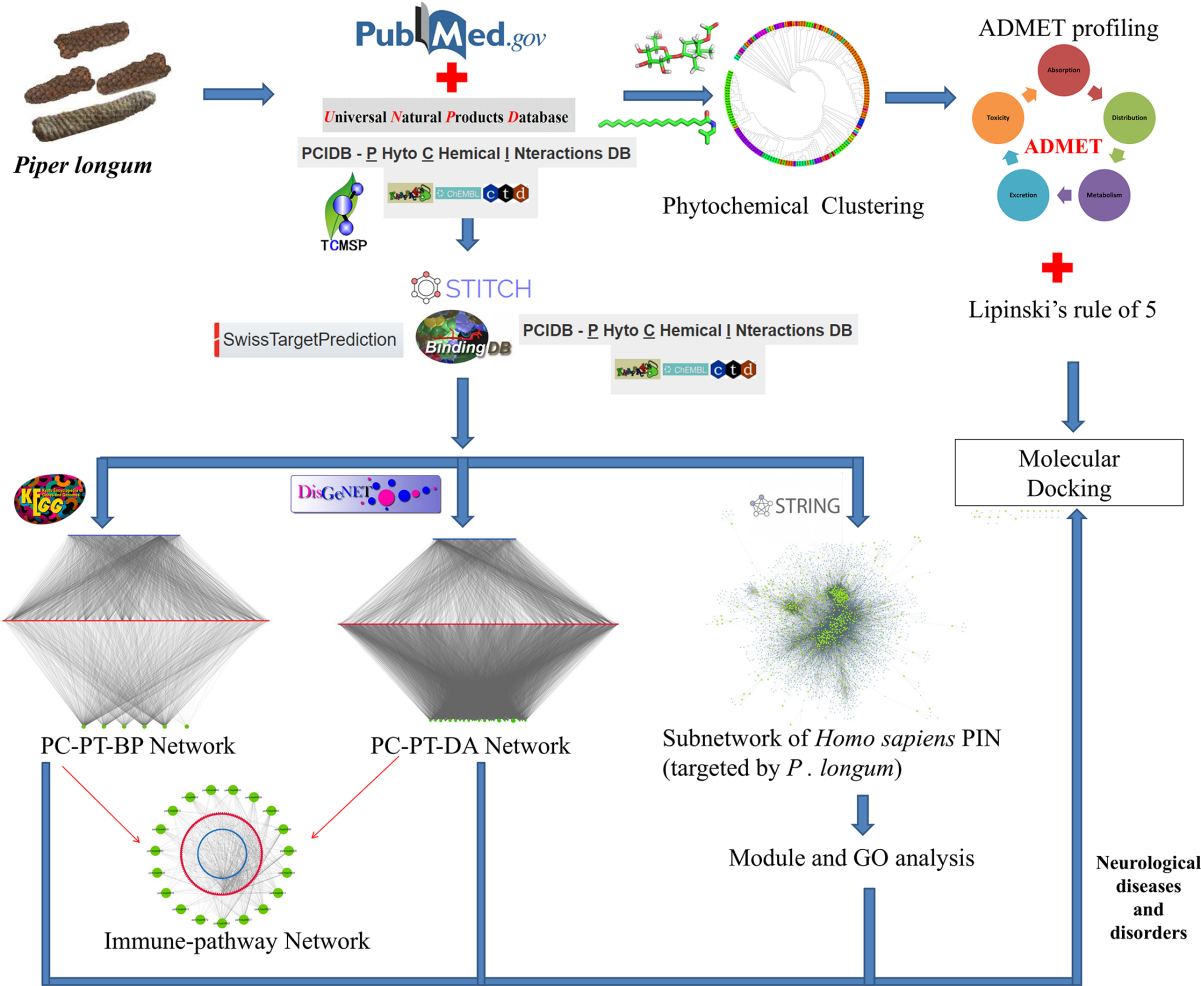
The workflow of this study.

In the present work, as the workflow (Fig 1), we firstly, reviewed the phytochemicals of *P*. *longum* as reported in the literature and public databases and attempted to cluster them in terms of their chemical and functional classes. Therapeutic relevance of these compounds was inferred through the network analysis of phytochemicals with their protein targets and their therapeutic activity was correlated with the number of proteins that a particular phytochemical may target. Further, the pharmacological action of these metabolites at biological level was explored and the potential metabolic and cellular pathways in which the target proteins are involved have been identified. We explain the disease association network that is constructed to interpret the relationship between the potential drug candidates in the human system. A subnetwork of human protein-protein interaction (PPI) network that is potentially regulated by *P*. *longum* was analyzed to identify functional modules present therein. Pharmaceutically relevant features of these phytochemicals were studied and drug likeliness of various phytochemicals was evaluated and finally, the molecular interactions of some of the potential drug-like phytochemicals with the protein targets involved in the neurological disorders were explored.

## Materials and methods

### Data collection

A dataset of phytochemicals present in *P*. *longum* was developed using extensive literature survey and mining of public database resources. Relevant research articles from PubMed-NCBI (https://www.ncbi.nlm.nih.gov/pubmed/) were selected and manually scrutinized. Three databases UNPD (Universal Natural Products Database) (pkuxxj.pku.edu.cn/UNPD) [21], TCMSP (Traditional Chinese Medicine Systems Pharmacology) [22] and PCIDB (PhytoChemical Interactions DB) (http://www.genome.jp/db/pcidb) [23] were screened for potentially active phytochemical present in *P*. *longum*. Chemical information of these phytochemicals was compiled from PubChem database [24] and ChEMBL [25] databases. Structural data of phytochemicals not available in PubChem and ChEMBL was derived using PubChem Sketcher v2.4 [26]. Compiled dataset was filtered to remove duplicate entries. Human proteins targeted by the phytochemicals were predicted from STITCH 5.0 (Search Tool for Interactions of Chemicals and proteins) [27], BindingDB [28], SwissTargetPrediction [29] and PCIDB.

The protein-chemical interaction reported in STITCH comes from the manually curated datasets including DrugBank [30] GLIDA [31], Matador [32], TTD (Therapeutic target database) [33] etc. In order to provide a full picture of the available data, STITCH also integrates information from several metabolic pathway databases and experimentally validated interactions from ChEMBL [25], PDB [34] and other sources. To access the high confidence targets, the interactions with a combined score of ≥0.4 were taken into account. The basic working principle of BindingDB is that similar compounds tend to bind the same proteins. It is a web-accessible database containing binding affinities of 20,000 (approx.) protein-ligand complexes. We screened the phytochemical-protein interactions having similarity search value ≥0.85. SwissTargetPrediction uses a combination of 2D and 3D similarity measures to identify the target proteins that can bind with a ligand showing the highest similarity with a library of 280,000 compounds. Top 15 protein targets for each phytochemical were selected from this resource. PCIDB returns a list of active compounds of the query herb and also enlists the possible genes involved in the interactions. We included all the phytochemical-protein interactions provided by PCIDB. UniProt IDs of all the protein targets identified from the above-mentioned four resources were used for network construction.

The biological pathways association of the identified protein targets was retrieved from KEGG database (Kyoto Encyclopedia of Genes and Genomes) [35]. A comprehensive platform of gene-disease association, called DisGeNETv4.0 [36] was used to find the disease information in which the protein targets may be involved. DrugBank database [37] was also mined to find-out the known target proteins.

### Compounds classification and clustering

An automated and rapid chemical classification method “ClassyFire” [38] was used to assign a chemical class to the phytochemicals. The query is mapped to the various classes based on its features that are calculated using superstructure-search operations and other properties. Clustering toolbox from ChemMine tools [39] was used for clustering the phytochemicals. The hierarchical clustering algorithm was opted, that forms a hierarchy of clusters based on pairwise compound similarities using the atom-pair descriptors and Tanimoto coefficient.

### Network construction and analysis

To investigate the pharmacological actions of the phytochemicals, various networks showing the interactions among phytochemical compounds (PC), protein targets (PT), biochemical pathways (BP) and associated diseases (AD) were constructed and analyzed using Cytoscape v3 [40].

### Modularity analysis of human PPI subnetwork

STRING v10.5 [41] was used to identify first-degree interactors of all the target proteins. Only high confidence interactions (score > = 0.9) were included to construct the PPI and duplicate edges were removed. MCL (Markov Cluster) algorithm [42] was used to cluster and organize the proteins in various modules. This algorithm detects cluster structure using mathematical bootstrapping procedure and has been shown that it is efficient in the identification of modules in PPI networks [43]. GO-based functional enrichment of these clusters was carried out using BINGO [44].

### Drug-likeliness prediction and molecular docking studies

Pharmacokinetic and toxicity properties of the compounds were studied using pKCSM which uses the graph-based structure signature method to predict a range of ADMET (Absorption, Distribution, Metabolism, Excretion and Toxicity) properties [45].

AutoDock 4.2 [46] was used to carry out the computational docking studies, using Lamarckian Genetic algorithm. The best binding orientation of the ligand within the protein cavity was estimated using binding energy values. The 2D representation of protein-ligand complexes for their molecular interactions was carried using Ligplot^+^ [47].

## Results and discussion

### Identification of phytochemicals

A dataset of phytochemicals present in *P*. *longum* was created from extensive literature survey and mining of natural product databases. In total 159 phytochemicals were identified and all the phytochemicals were assigned a unique ID. Details of all the phytochemicals i.e. their unique IDs, names and their references are presented in Table 1.

**Table 1:** List of phytochemicals identified in *P*. *longum*.

S. No.	Phytochemical ID	Phytochemical name	Pubchem/ ChEMBL ID	Reference
1	PL1	3β, 4α-dihydroxy-1-(3-phenylpropanoyl)-piperidine-2-one	N/A	[48]
2	PL2	(2E, 4E, 14Z)-6-hydroxyl-N-isobutyleicosa-2,4,14-trienamide	N/A	[48]
3	PL3	Coumaperine	10131321	[49]
4	PL4	N-5-(4-hydroxy-3-methoxyphenyl)-2E-pentenoyl piperidine	N/A	[49]
5	PL5	Piperolactam A	3081016	[49]
6	PL6	1-[1-oxo-5 (3,4-methylenedioxyphenyl) -2E,4E-pentadienyl]–pirrolidine	N/A	[49]
7	PL7	(R)-(-)–turmerone	558221	[49]
8	PL8	Octahydro-4-hydroy-3alpha-methyl-7-methylene-alpha-(1-methylethyl)-1H-indene-1-methanol	N/A	[49]
9	PL9	(+) -aphanamol I	11031884	[49]
10	PL10	Bisdemethoxycurcumin	5315472	[49]
11	PL11	Demethoxycurcumin	5469424	[49]
12	PL12	Longumosides A	N/A	[50]
13	PL13	Longumosides B	71579641	[50]
14	PL14	Erythro-1-[1-oxo-9(3,4-methylenedioxyphenyl)-8,9-dihydroxy-2E-nonenyl]-piperidine	N/A	[50]
15	PL15	Threo-1-[1-oxo-9(3,4-methylenedioxyphenyl)-8,9-dihydroxy-2E-nonenyl]-piperidine	N/A	[50]
16	PL16	3β,4α-dihydroxy-2-piperidinone	N/A	[50]
17	PL17	5,6-dihydro-2(1H)-pyridinone	N/A	[50]
18	PL18	Piperlongumide (1) [N-isobutyl-19-(3′,4′-methylenedioxyphenyl)-2E,4E nonadecadienamide]	N/A	[51]
19	PL19	1-(3,4-methylenedioxyphenyl)-1E tetradecene	N/A	[51]
20	PL20	Piperlongimin A [2E-N-isobutyl-hexadecenamide]	N/A	[51]
21	PL21	2E,4E-N-isobutyl-octadecenamide	N/A	[51]
22	PL22	Piperlongimin B [2E-octadecenoylpiperidine]	N/A	[51]
23	PL23	2E,4E-N-isobutyl-dodecenamide	N/A	[51]
24	PL24	2E,4E,12E,13-(3,4-methylenedioxyphenyl)-trideca-trienoic acid isobutyl amide	N/A	[51]
25	PL25	Piperine	638024	[51]
26	PL26	Pellitorine	5318516	[52]
27	PL27	N-[(2E,4E)-Decadienoyl]-piperidine	11118018	[52]
28	PL28	N-Isobutyl-2E,4E-undecadienamide	20157325	[52]
29	PL29	Piperlonguminine	5320621	[52]
30	PL30	Piperanine	5320618	[52]
31	PL31	N-[(2E,4E)-Tetradecadienoyl]piperidine	11130083	[52]
32	PL32	N-Isobutyl-2E,4E-hexadecadienamide	6442402	[52]
33	PL33	Pipercallosine	5372201	[52]
34	PL34	(2E,4E,12Z)-N-Isobutyl-octadeca-2,4,12-trienamide	N/A	[52]
35	PL35	N-Isobutyl-2E,E-octadecadienamide	9974234	[52]
36	PL36	Dehydropipernonaline	6439947	[52]
37	PL37	Pipernonatine	9974595	[52]
38	PL38	(E)-9-(Benzo[d][1,3]dioxol-5-yl)-1-(piperidin-1-yl)non-2-en-1-one	N/A	[52]
39	PL39	1-(2E,4E,12E)-Octadecatrinoylpiperidine	N/A	[52]
40	PL40	Retrofractamide B	5372162	[52]
41	PL41	(2E,4E,14Z)-N-Isobutyleicosa-2,4,14-trienamide	N/A	[52]
42	PL42	N-isobutyl-2E,4E-decyldecadienamide	N/A	[52]
43	PL43	(2E,4E,10E)-N-11-(3,4-Methylenedioxyphenylhmdecatrienoylpiperidine	N/A	[52]
44	PL44	1-[(2E,4E,14Z)-1-Oxo-2,4,14-eicosatrienyl]-piperidine	N/A	[52]
45	PL45	Guineensine	6442405	[52]
46	PL46	(2E,4E,14Z)-N-Isobutyldocosa-2,4,14-trienamide	N/A	[52]
47	PL47	(2E,4E,12E)-13-(Benzo[d][1,3]dioxol-6-yl)-1-(piperidin-1-yl)trideca-2,4,12-trien-1-one	N/A	[52]
48	PL48	(2E,4E,13E)-14-(Benzo[d][1,3]dioxol-6-yl)-N-isobutyltetradeca-2,4,13-trienamide	N/A	[52]
49	PL49	Brachyamide B	14162526	[52]
50	PL50	Dihydropiperlonguminine	12682184	[52]
51	PL51	Piperdardine	10086948	[52]
52	PL52	Retrofractamide A	11012859	[52]
53	PL53	Piperchabamide D	16041827	[52]
54	PL54	N-isobutyl-2E,4E-dodecadienamide	6443006	[52]
55	PL55	Piperchabamide B	44453655	[52]
56	PL56	13-(1,3-Benzodioxol-5-yl)-N-(2-methylpropyl)-(2E,4E)-tridecadienamide	N/A	[52]
57	PL57	Piperchabamide C	44454018	[52]
58	PL58	1-[(2E,4E)-1-oxo-2,4-hexadecadienyl]-piperidine	10980124	[52]
59	PL59	2,2-Dimethoxybutane (C6H14O2]	137941	[53]
60	PL60	2-Hydroxy myristic acid (C14H28O3]	1563	[53]
61	PL61	β-Myrcene (C10H16]	31253	[53]
62	PL62	N-methyl-1- octadecanamine (C19H41N]	75539	[53]
63	PL63	Piperazine adipate (C10H20N2O4]	8905	[53]
64	PL64	2-Nonynoic acid (C9H14O2]	61451	[53]
65	PL65	Dodecanal (CH3(CH2]10CHO]	8194	[53]
66	PL66	1,2-Benzenedicarboxylic acid, bis(2-ethylhexyl] ester (C24H38O4]	8343	[53]
67	PL67	2-Amino-4-hydroxypteridine-6-carboxylic acid (C7H5N5O3]	70361	[53]
68	PL68	Piperlongumine	637858	[54]
69	PL69	Hydrocinnamic acid (HCI)	107	[22]
70	PL70	Palmitic acid	985	[22]
71	PL71	1,8-cineole	2758	[22]
72	PL72	Lawsone	6755	[22]
73	PL73	Cis-Decahydronaphthalene	7044	[22]
74	PL74	Piperonylic acid	7196	[22]
75	PL75	Hypnon	7410	[22]
76	PL76	Moslene	7461	[22]
77	PL77	Cymol	7463	[22]
78	PL78	Methyl hydrocinnamate	7643	[22]
79	PL79	Hexahydropyridine (PIP)	8082	[22]
80	PL80	Pisol	8193	[22]
81	PL81	Piperonal	8438	[22]
82	PL82	Isobutylisovalerate	11514	[22]
83	PL83	Tridecane (TRD)	12388	[22]
84	PL84	Pentadecane (MYS)	12391	[22]
85	PL85	N-Heptadecane	12398	[22]
86	PL86	N-Nonadecane (UPL)	12401	[22]
87	PL87	Tridecylene	17095	[22]
88	PL88	Heptadecene	23217	[22]
89	PL89	Pentadecene	25913	[22]
90	PL90	Nonadecene	29075	[22]
91	PL91	tetradecadiene-1,13	30875	[22]
92	PL92	Linalool (D)	67179	[22]
93	PL93	Cyclopentadecane	67525	[22]
94	PL94	Beta-Bisabolene	68128	[22]
95	PL95	Sesamol	68289	[22]
96	PL96	Sesamin	72307	[22]
97	PL97	p-Amino-o-cresol	76081	[22]
98	PL98	2,4-Dimethoxytoluene	96403	[22]
99	PL99	D-Camphor (CAM)	159055	[22]
100	PL100	Cis-2-Decalone	246289	[22]
101	PL101	Piperitenone	381152	[22]
102	PL102	(-)-Nopinene	440967	[22]
103	PL103	(-)-Alpha-Pinene	440968	[22]
104	PL104	Isodiprene (CHEBI:7)	443156	[22]
105	PL105	(R)-linalool	443158	[22]
106	PL106	N-(2,5-dimethoxyphenyl)-4-methoxybenzamide	532276	[22]
107	PL107	Anethole	637563	[22]
108	PL108	Isoeugenol	853433	[22]
109	PL109	(3S)-3,7-dimethylocta-1,6-dien-3-yl] propanoate	1616358	[22]
110	PL110	()-Terpinen-4-ol	2724161	[22]
111	PL111	Alpha-Farnesene	5281516	[22]
112	PL112	Farnesene	5281517	[22]
113	PL113	alpha-humulene	5281520	[22]
114	PL114	Isocaryophyllene	5281522	[22]
115	PL115	p-Ocimene	5281553	[22]
116	PL116	8-Heptadecene	5364555	[22]
117	PL117	9,17-Octadecadienal (Z)	5365667	[22]
118	PL118	Cyclodecene, 1-methyl-	5367581	[22]
119	PL119	1,4,7,-Cycloundecatriene, 1,5,9,9-tetramethyl-, Z,Z,Z-	5368784	[22]
120	PL120	(+/-)-Isoborneol	6321405	[22]
121	PL121	(Z)-caryophyllene	6429301	[22]
122	PL122	cis-.beta.-Elemene diastereomer	6431152	[22]
123	PL123	N-Isobutyl-2,4-icosadienamide	6441067	[22]
124	PL124	(E,E,E)-11-(1,3-Benzodioxol-5-yl)-N-(2-methylpropyl)-2,4,10-undecatrienenamide	6453083	[22]
125	PL125	Epieudesmin (ZINC03996196)	7299790	[22]
126	PL126	Valencene	9855795	[22]
127	PL127	(1S,5S)-1-isopropyl-4-methylenebicyclo[3.1.0]hexane	11051711	[22]
128	PL128	(5S)-5-[(1R)-1,5-dimethylhex-4-enyl]-2-methylcyclohexa-1,3-diene	11127403	[22]
129	PL129	(E)-5-(4-hydroxy-3-methoxy-phenyl)-1-piperidino-pent-2-en-1-one	11630663	[22]
130	PL130	(3R,8S,9S,10R,13R,14R,17R)-17-[(2R,5S)-5-ethyl-6-methylheptan-2-yl]-10,13-dimethyl-2,3,4,7,8,9,11,12,14,15,16,17-dodecahydro-1H-cyclopenta[a]phenanthren-3-ol (ZINC03982454)	11870467	[22]
131	PL131	Delta-elemene	12309449	[22]
132	PL132	(2R,4aR,8aR)-2-methyldecalin	12816526	[22]
133	PL133	(1R,5R,7S)-4,7-dimethyl-7-(4-methylpent-3-enyl)bicyclo[3.1.1]hept-3-ene	13889654	[22]
134	PL134	Calarene	15560279	[22]
135	PL135	Bisdemethoxycurcumin	45934475	[22]
136	PL136	1,4-cadinadiene	50986185	[22]
137	PL137	Tricyclene	55250308	[22]
138	PL138	Alpha-Cubebene	42608159	[22]
139	PL139	Piperundecalidine	44453654	[22]
140	PL140	3-phenylundecane	20655	[22]
141	PL141	4-[(1-Carboxy-2-methylbutyl)amino]-2(1H)-pyrimidinone	591989	[22]
142	PL142	Bicyclo[3. 2. 2]non-6-en-3-one	N/A	[22]
143	PL143	Cedryl acetate	9838172	[22]
144	PL144	Isolongifolene epoxide	107035	[22]
145	PL145	N-isobutyleicosa-2(E),4(E),8(Z)-trienamide	N/A	[22]
146	PL146	Pisatin	101689	[22]
147	PL147	Tetradecahydro-1-methylphenanthrene	609802	[22]
148	PL148	Undulatone	5281311	[22]
149	PL149	Copaene	25245021	[22]
150	PL150	Linalool	6549	[22]
151	PL151	Sylvatine	N/A	[22]
152	PL152	beta-Cubebene	93081	[22]
153	PL153	(-)-Caryophyllene oxide	1742210	[22]
154	PL154	(-)-alpha-cedrene	6431015	[22]
155	PL155	(+)-Fargesin	CHEMBL462822	[23]
156	PL156	Piperolactam A	CHEMBL387864	[23]
157	PL157	(+)-Sesamin	CHEMBL252915	[23]
158	PL158	2-Phenylethanol	CHEMBL448500	[23]
159	PL159	l-Zingiberene	CHEMBL479020	[23]

### Compounds classification and clustering

From the chemical classification, 159 phytochemicals were found to be distributed among 26 different classes of compounds. Out of these, 11 classes contain only one phytochemical and these correspond to carboxylic acids and derivatives (PL141), cinnamic acids and derivatives (PL68), isoflavonoids (PL146), naphthalenes (PL72), organic nitrogen compounds (PL62), oxanes (Pl71), phenanthrenes and derivatives (PL147), phenol ethers (PL107), phenylpropanoic acids (PL69), pteridines and derivatives (PL67), pyridines and derivatives (PL17) and steroid and its derivatives (PL130). Adaptation of plants against various abiotic and biotic stresses over millions of years of evolution is responsible for such chemical diversity of the phytochemicals [55]. Among all these classes, the benzodioxole group constitutes the highest number of phytochemicals (31) and they are found to be clustered together. It is a widely dispersed class of compounds among natural as well as synthetic drugs [56].

**Fig 2:**
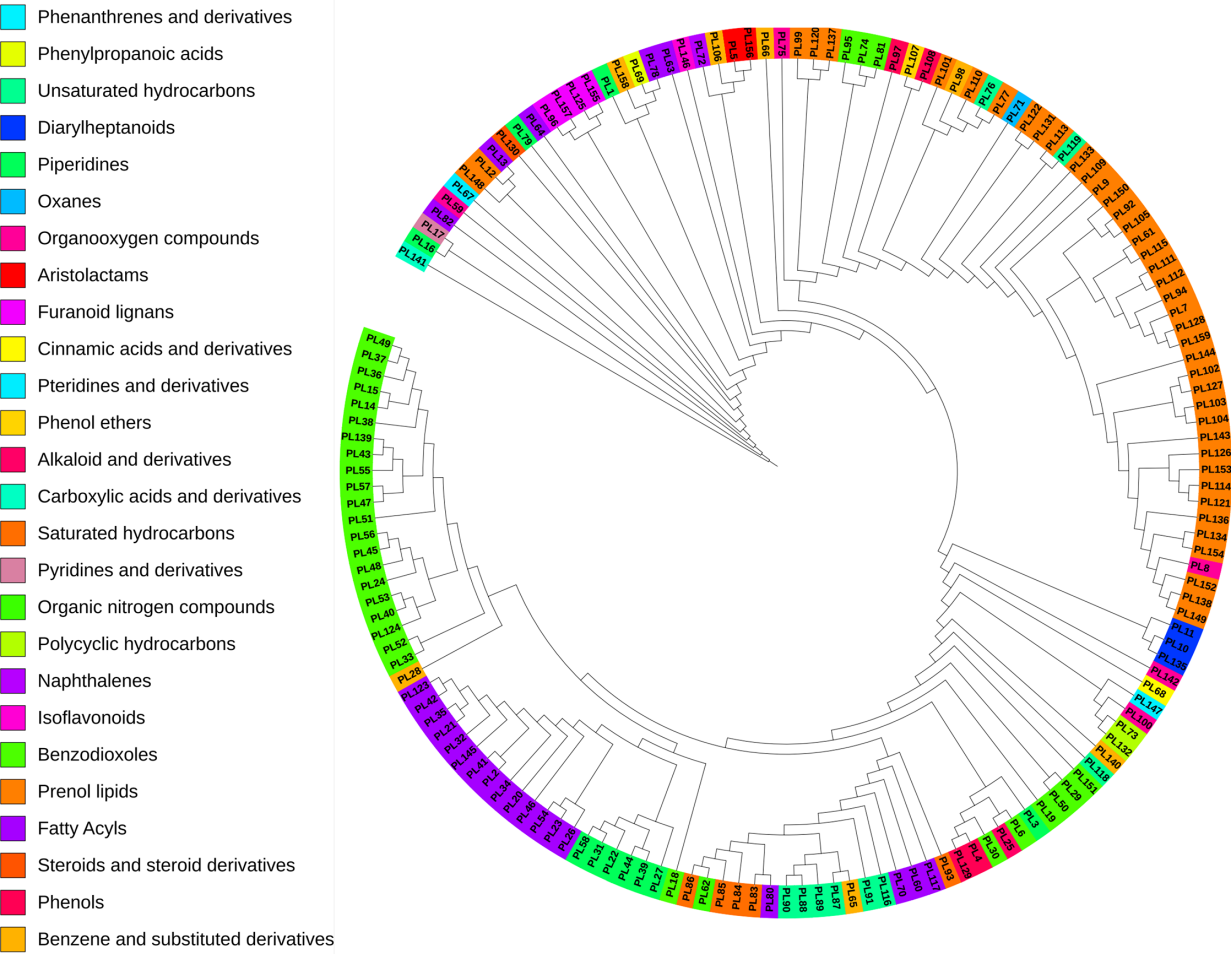
Hierarchical clustering of phytochemicals belonging to *Piper longum*. Phytochemicals were clustered on the basis of atom-pair descriptors and Tanimoto coefficient using Chemmine tool. It can be easily seen that most of the phytochemicals belonging to benzodioxoles, fatty acyl and prenol lipids category were clustered together.

The hierarchical clustering of phytochemicals is shown in Fig 2. The phylogenetic tree reveals that phytochemicals cluster with molecules that share similar scaffold. The class of chemical compounds corresponding to prenol lipids and fatty acyls are highly prominent in the dataset; a good agreement with the known fact that lipids form a large group of primary metabolites of the plant [57].

### Phytochemical–protein target (PC-PT) network

**Fig 3:**
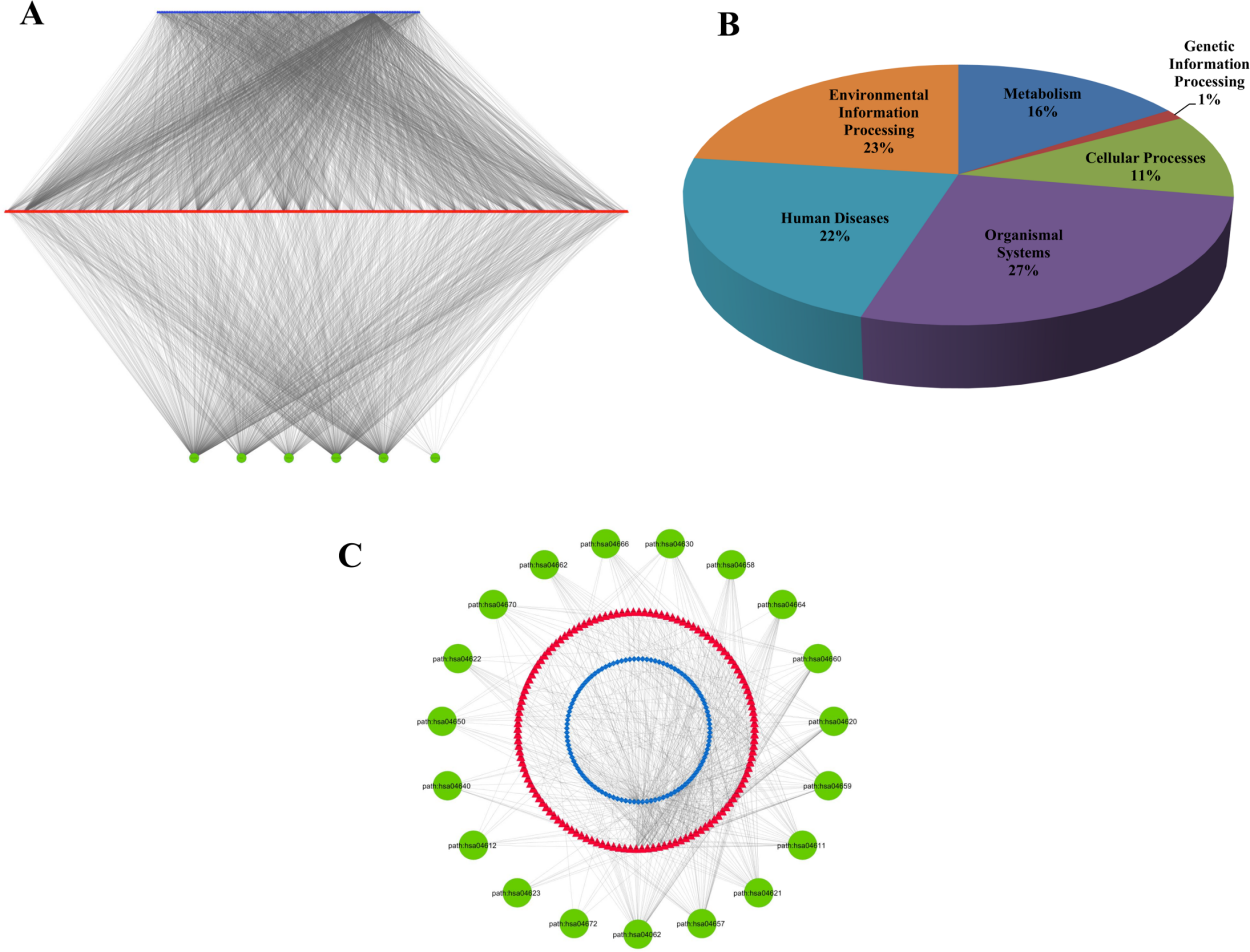
(A). A tripartite phytochemical—protein target—biochemical pathway (PC-PT-BP) network of the constituents of *Piper longum*. Top layer (blue) represents phytochemicals (159), middle layer (red) represents the potential protein targets (1109) and the third layer (green) represents association of protein targets with six pathway classes: metabolism, genetic information processing, environment information processing, cellular processes, organismal systems and human diseases. (B) Involvement of target proteins of *Piper longum* in various human pathway classes. The human pathway mapping of identified target proteins were distributed among 6 classes. Genetic information processing class includes pathways belonging to transcription, translation, replication & repair, folding, sorting and degradation. Environment information processing includes membrane transport, signal transduction, signaling molecules and their interactions. Cellular processes involve pathways of cell growth, cell death and transport (endocytosis, phagosome, lysosome etc.). Organismal system includes immune, endocrine, circulatory, digestive, excretory, nervous, sensory system. Human diseases and metabolism include pathways associated with diseases and metabolic system (carbohydrates, lipids, amino acids etc.) respectively. (C) Bioactives of *Piper logum* affecting the Human immune system. The first layer (blue) represents 106 phytochemicals, regulating 11 immune pathways (green) by targeting 131 proteins (red).

**Fig 4:**
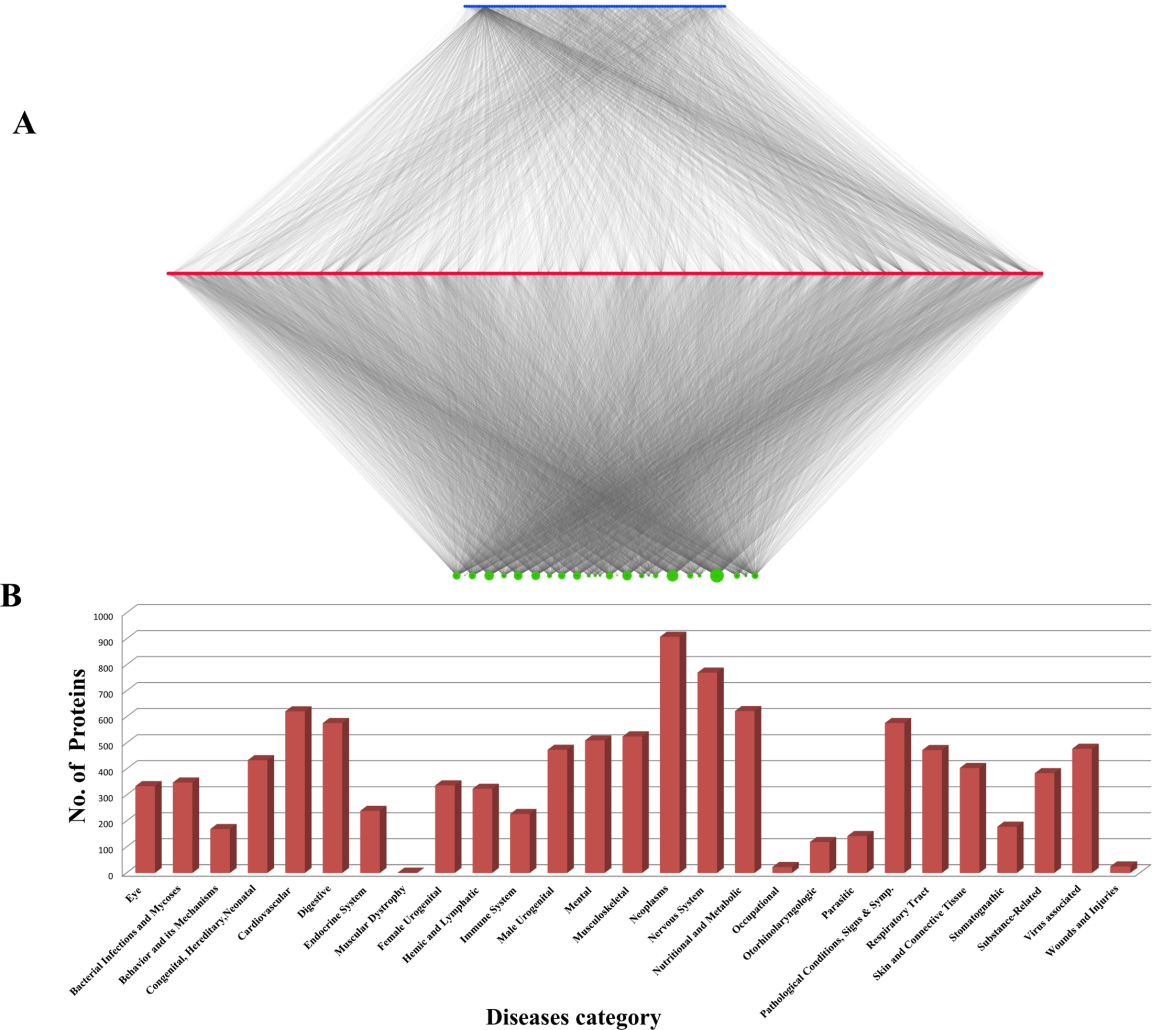
(A). Phytochemical—protein target—disease association (PC-PT-DA) network of *Piper longum*. Top layer (blue) represents phytochemicals **(159)**, middle layer (red) represents their protein targets **(1109)** and the bottom layer (green) represents association of protein targets with 27 disease classes obtained from DisGeNET. Size of the nodes in third layer (green) is proportional to their degree value. (B) *Piper longum* protein targets and their involvement in 27 disease classes. Number of potential protein targets associated with various disease classes as obtained from DisGeNETv4.0. Maximum number of targets are related with neoplasm and nervous system, while muscular dystrophy and occupational diseases have least number of targets associated with them.

For understanding the interactions between small molecules and proteins, the PC-PT bipartite network was constructed by mapping 159 phytochemicals to their potential proteins targets. This resulted in the identification of 1109 unique human proteins that may be potentially targeted by the phytochemicals of *P*. *longum*. As may be seen in top two layers of the tripartite network in Figs 3A and 4A, many phytochemicals are found to interact with multiple proteins, an effect known as polypharmacology. Polypharmacological effect of the *P*. *longum* phytochemicals was evaluated using its PCt/Tt value (the average value of a number of targets for each compound). This value is calculated for each phytochemical identified, higher PCt/Tt value for a compound suggests that it may be an activator or an inhibitor of multiple proteins and may be individually or in combination serve as lead-compound. Numerous existing drugs are well known for their multi-targeting activities. One such example is Aspirin; which is usually used as an analgesic and at times also as an antipyretic [58], and its anti-inflammatory medication in treatment of various diseases like rheumatoid arthritis [59], pericarditis [60] is also well known.

PL70 (palmitic acid) being ubiquitous in nature emerges out as a compound with the highest PCt/Tt value (0.55) in the network. Next in order are the phytochemicals PL99, PL11, PL25 and PL66 with PCt/Tt value 0.068, 0.060, 0.057, 0,052 respectively. Network analysis shows that 99% of these phytochemicals are linked with more than 10 protein targets, indicating towards the multi-target properties of this herb. However, sometimes the concept of polypharmacology could be a double-edged sword and may cause adverse effects when the idea is not fully understood [61]. In that case, the approach may result in identifying off-targets for a new drug.

We also explored the specificity of these phytochemicals to hit a unique target. Among 159 phytochemicals, PL59 (2, 2-Dimethoxybutane (C6H14O2)) is one such compound, that is shown to interact with a single target (UniProt ID—P08575). P08575 (receptor-type tyrosine-protein phosphatase C, CD45) plays a critical role in receptor-mediated signaling in both B and T-cells [62], [63]. Since altered signaling process is one of the causes that leads to several disorders including SCID (Severe combined immunodeficiency syndrome), in such cases the explicit behavior of PL59 and its reliability to target specific protein may be evaluated.

Among 159 phytochemicals, 109 target P10636. Such a high degree corresponding to this node refers its ability to interact with other phytochemicals. In other words, it is the most commonly targeted protein in the network. P10636 is a “microtubule-associated protein tau (MAPT)” encoded by the MAPT gene. A system level investigation of neurodegenerative dementia reveals the accumulation of this protein in a diseased state, including frontotemporal dementia and supranuclear palsy [64]. Hence, it can be assumed that these 109 phytochemicals may contribute to the *P*. *longum*’s effect in the treatment of mental diseases. Although previous studies on treatment of mental disorders by the herbal extracts are known [12], piperine (PL25) and piperlongumine (PL68) have got special attention against Parkinson’s disease [65]. Detailed study on the pharmacophore properties of additional phytochemicals identified in *P*. *longum* will help in deciphering its detailed mechanism and highlighting their key features that may be helpful in designing synthetic drugs. Additionally, the positive synergistic effect of the compounds can be explored for better affinity and efficacy.

Other proteins with high degree centrality include Q9NUW8, Q9NR56, Q5VZF2, Q9NUK0, P22303 and P06276 with a value corresponding to 64, 60, 59, 57, 56 and 55 respectively. These proteins may be given special attention in aspects of behaving as key targets, specifically in light of the fact that interventions at specific proteins can be weak in terms of binding affinity, yet they may be highly effective in combinations [66].

### Phytochemical, protein target and biochemical pathway (PC-PT-BP) network

To obtain a global view of pathways targeted by *P*. *longum*, a tripartite network was constructed using its phytochemicals, their protein targets and associated biochemical pathways Fig 3A. 279 unique human pathway maps were classified into 6 broad categories i.e. metabolism, genetic information processing, environmental information processing, cellular processes, organismal systems and human diseases. The detailed mapping of target proteins into different pathways is given in S1 Table.

As suggested in Fig 3B, the highest numbers of target proteins (455, 27%) are associated with pathways belonging to organismal systems, followed by environmental information processing category (384, 23%) and human diseases (371, 22%). These findings suggest that the mode of action of these phytochemicals may be largely *via* regulating organismal systems (which include immune, endocrine, circulatory, digestive, nervous and excretory system). An earlier *in vivo* study on *P*. *longum* also support this finding, in which it was shown that immunomodulatory properties of the herbal extract lead to increase in white blood cells (WBC) in Balb/c mice [9]. The endocrine effect is explained *via* the process of ovulation which includes interrelationship between the endocrine and cytokine system. By modulating the inflammatory mediators like cytokines, reactive oxygen species etc., phytochemicals of this plant exert their antifertility properties [67]. Similarly, its effect on cardiovascular [15], digestive [68], [69], nervous [12], [65] and excretory [70] systems has also been studied. Protein targets such as P28482, Q9Y243, P31751, P31749, P42336, P27986, and P27361 are found to be involved in many pathways. Thus it may be hypothesized that these proteins may be important targets, as their modulation may lead to regulation of multiple pathways.

To explore the basic principle of the herb in relation to the human immune system, a sub-network of immune pathways being regulated by *P*. *longum* was created. 106 phytochemicals out of 159 are shown to regulate 19 human immune pathways *via* 131 proteins. 57.25% of these proteins are affecting the immune system *via* chemokine (hsa04062) and interleukin (hsa04657) signaling pathways. The role of piperine in reducing Th2 cytokines and regulating cytokine in asthma models have been explored earlier [71].

This sub-network identifies Prostaglandin G/H synthases (PTGS) as common targets. PTGS-1 (P23219) and PTGS-2 (P35354) are the targets of 28 and 22 phytochemicals respectively. This enzyme is involved in the conversion of arachidonate to prostaglandin H2, which are the major components that induce inflammation and pain [72]. The high rate of edema inhibition by herb-oil in comparison to the standard anti-inflammatory drug ‘Ibuprofen’ is also reported [73].

Further, the network data provides that NOD-like receptors and Toll-like receptors (TLRs) are regulated through 33 and 29 targets respectively. The receptors play a key role in innate immunity. The pathogen invasion caused by bacterial lipopolysaccharide (LPS) induces signaling pathways which further lead to the activation of macrophages *via* TLRs. An earlier report on the herb also shows that the root area possesses anti-amoebic properties [69]. Thus, to explore the effect of this herb on the innate immunity, the sequence of events which lead to the interaction of receptor proteins with these phytochemicals (especially present in root region) may be specifically focused upon. Various studies have supported the anti-inflammatory behavior of *P*. *longum*. Our work reveals that this effect is not due to a limited number of phytochemicals rather a vast number of phytochemicals are involved in this property. Among 105 phytochemicals involved in immunomodulation, palmitic acid (PL70), demethoxycurcumin (PL11), bisdemethoxycurcumin (PL10), 1,2-benzene dicarboxylic acid (PL66), sesamin (PL96) and piperine (PL25) are the top-immunomodulators with 76, 15,12, 11, 11, 10 protein targets, respectively. Thus, combining the effect of other phytochemicals reported in our study will help to provide a wholistic view of the herb’s immunomodulatory potency. Additionally, the use of analytical and structural chemistry of the phytochemicals and phytochemical-protein target complexes will help in understanding the molecular mechanism in detail. The detailed information of the immune pathways considered and the number of target proteins involved in each class is presented in Table 2.

**Table 2:** List of protein targets of *P*. *longum’s* biochemicals involved in immune pathways of *Homo sapiens*.

Immune pathway	Immune pathway ID	Number of protein targets involved	Uniprot IDs of protein targets involved
Chemokine signaling pathway	path:hsa04062	41	P31749, O14920, P10145, P19838, P49840, P49682, Q9NQ66, O00574, Q15147, Q01970, Q00722, P62873, P59768, P05771, P51686, P51681, P51679, P51677, O43524, P46094, P42336, Q04206, P41597, P61073, P49841, P28482, P34947, P32246, P08754, P04899, P27986, P27361, Q9Y243, P25098, P09769, Q05655, P40763, P31751, P25963, P13500, P12931.
Platelet activation	path:hsa04611	30	P12931, P31749, Q9NQ66, Q16539, Q15759, O15264, Q96RI0, Q15147, P24557, P23219, Q01970, Q00722, P47712, P29474, P53778, P50148, P47900, P43119, P42336, P63092, P28482, P08754, P04899, P27986, P27361, Q9Y243, P25116, P06241, P21731, P31751.
Antigen processing and presentation	path:hsa04612	9	P01375, Q13952, P07858, P61769, P07711, P25774, P25208, P23511, P16220,
Toll-like receptor signaling pathway	path:hsa04620	29	P31749, O14920, P10145, P19838, P05231, P01584, P01375, Q16539, O60603, Q15759, O15264, P53779, O00206, P45984, P45983, Q14790, P53778, P05412, P42336, Q04206, P28482, P27986, P01100, P27361, P43235, Q9Y243, Q9NR96, P31751, P25963.
NOD-like receptor signaling pathway	path:hsa04621	33	O14920, P10415, P10145, P19838, P05231, P01584, P01375, Q9NQ66, Q16539, Q9H3M7, Q15759, O15264, P53779, O00429, O00206, Q5T6X5, P45984, Q15147, P45983, P07858, Q14790, Q01970, Q00722, P53778, P05412, Q9Y5S1, Q04206, P41180, P28482, P27361, Q05655, P13500, P25963.
RIG-I-like receptor signaling pathway	path:hsa04622	14	O14920, P10145, P19838, P01375, Q16539, Q15759, O15264, P53779, P45984, P45983, Q14790, P53778, Q04206, P25963.
Cytosolic DNA-sensing pathway	path:hsa04623	6	O14920, P19838, P05231, P01584, Q04206, P25963.
Jak-STAT signaling pathway	path:hsa04630	17	P31749, P10415, P60568, P01106, P05231, Q92793, P22301, P17706, P42345, P42336, P27986, P04141, Q9Y243, Q09472, P24385, P40763, P31751.
Hematopoietic cell lineage	path:hsa04640	10	P15144, P05231, P01584, P01375, P04141, P08473, P07333, P16671, P15813, P15812.
Natural killer cell mediated cytotoxicity	path:hsa04650	13	P06239, P01375, P42574, P17252, P05771, P05129, P42336, O14763, P28482, P27986, P04141, P27361, P06241.
IL-17 signaling pathway	path:hsa04657	34	O14920, P10145, P19838, P14780, P05231, P01584, P01375, Q16539, Q15759, O15264, P53779, Q8TD08, P45984, Q16659, P45983, P35354, Q13164, Q14790, P45452, P08254, P03956, P42574, P53778, P17535, P05412, Q04206, P49841, P28482, P31152, P04141, P01100, P27361, P13500, P25963.
Th1 and Th2 cell differentiation	path:hsa04658	18	O14920, P60568, P19838, P06239, Q16539, Q15759, O15264, P53779, P45984, P45983, P53778, P05412, Q04206, P28482, P01100, P27361, Q04759, P25963.
Th17 cell differentiation	path:hsa04659	29	O14920, P60568, P19838, P06239, P05231, P01584, Q16539, Q15759, O15264, P53779, P45984, P45983, P53778, P42345, P05412, Q04206, P51449, P35869, Q16665, P35398, P84022, P28482, P01100, P27361, P19793, Q04759, P36897, P40763, P25963.
T cell receptor signaling pathway	path:hsa04660	28	P31749, O14920, P60568, P19838, P06239, P01375, Q16539, Q15759, O15264, P45984, P22301, P53778, P08575, P11802, P05412, P42336, Q04206, P49841, P28482, P27986, P04141, P01100, P27361, Q9Y243, P06241, Q04759, P31751, P25963.
B cell receptor signaling pathway	path:hsa04662	16	P31749, O14920, P19838, O15357, P05771, P05412, P42336, Q04206, P49841, P28482, P27986, P01100, P27361, Q9Y243, P31751, P25963.
Fc epsilon RI signaling pathway	path:hsa04664	20	P31749, P01375, Q16539, Q15759, O15264, P53779, P45984, P45983, P09917, P47712, P17252, P53778, P42336, P28482, P27986, P04141, P27361, Q9Y243, P06241, P31751.
Fc gamma R-mediated phagocytosis	path:hsa04666	17	P31749, P50570, O15357, P47712, P17252, P05771, P05129, P08575, P42336, P28482, P27986, P27361, Q9Y243, P23443, Q05655, Q02156, P31751.
Leukocyte transendothelial migration	path:hsa04670	15	P08253, P14780, Q16539, Q15759, O15264, P17252, P53778, P05771, P05129, P42336, P61073, P35222, P08754, P04899, P27986.
Intestinal immune network for IgA production	path:hsa04672	5	P60568, P05231, P22301, P51686, P61073.

### Phytochemical, protein target and disease association (PC-PT-DA) network

The data of protein targets and the diseases in which they are involved was collected and a tripartite network were drawn (Fig 4A). The diseases were classified into 27 classes and distribution of proteins among each of these classes is shown in Fig 4B. It can be easily seen that majority of protein targets have their association with neoplasm and nervous system diseases, that are 908 and 770 proteins, respectively. Numerous studies have shown the effect of long pepper plant in the treatment of different cancers including prostate, breast, lung, colon, lymphoma, leukemia, primary brain tumors and gastric cancer. A recent study states that the plant’s anti-cancerous property is due to the inhibitory mechanism of Glutathione S-transferase pi 1 (GSTP1) by its compound piperlongumine (PL29). GSTP1 is overexpressing protein in cancerous cells and the reactive olefins in the piperine (PL25) attenuate the cancer-cell proliferation by blocking its active site [74].

According to the network data, the association between *P*. *longum* and nervous system diseases is mainly by the regulation of 434 protein targets. Among these, the majority of proteins are involved in signaling transmission and developmental pathways. Out of total 434, 29 protein targets are found to be interacting with piperine (PL25) that is known to have anti-epileptic [75], analgesic and anti-convulsant [76] nature. Anti-depression like activity of this compound on animal samples suggests that the compound may act as a potential functional food to improve brain function [77]. Analysis of degree distribution shows that 1,2-Benzenedicarboxylic acid (PL66), demethoxycurcumin (PL11) and D-camphor (PL99) are having a higher number of protein interactors in comparison to piperine (PL25) i.e 36, 34 and 33, respectively. Thus, it may be inferred that these phytochemicals may also have an effect in neuroprotection and may act as new lead compounds for neurodegenerative disorders.

**Fig 5:**
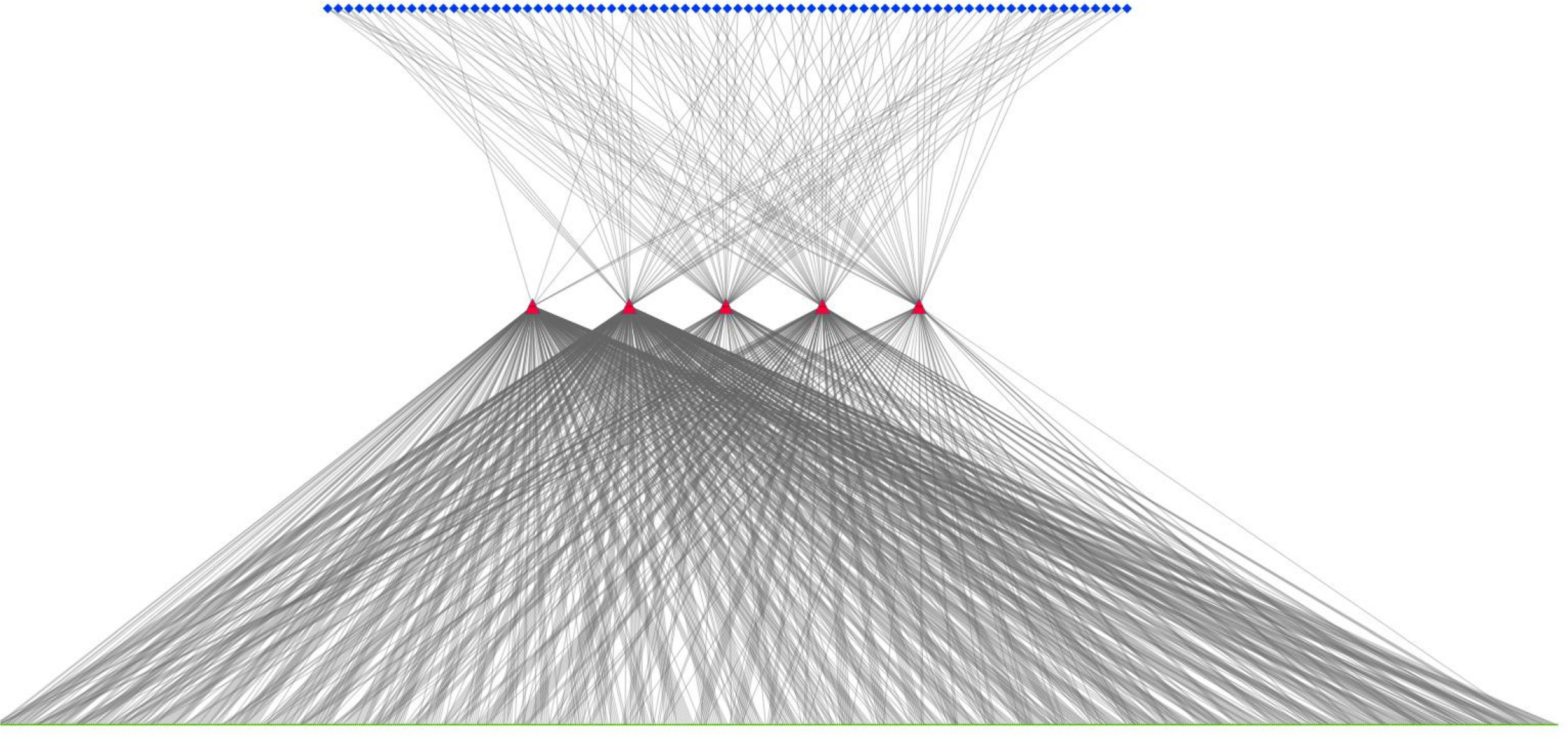
Potential FDA-approved targets from target dataset of *Piper longum*. Middle layer (red) represents the 5 key protein targets, which are regulated by 77 phytochemicals of *Piper longum*, represented in the top layer (blue). Third layer (green) represents the mapping of these targets in 825 diseases.

The prioritization of the target proteins of *P*. *longum* was performed by mapping them to the approved drug targets of DrugBank with the intent of selecting known drug targets for identifying their novel regulators from this herb’s constituents. For this, common target proteins among all the four target prediction software were selected. Five proteins (P04150, P37231, Q8NER1, P21397, and P27338) among these are FDA approved drug-targets, reflecting their importance for the identification of their novel regulators. The position of these potential targets in the PC-PT-DA network shows that 77 phytochemicals are involved in their regulation (Fig 5). Further, these key targets are associated with a diverse array of diseases that means they are involved at multiple levels in the biological system. Twenty eight protein targets were predicted by three of the four softwares used for phytochemical-protein interaction prediction. These may be considered as potential targets and may be further explored with respect to the phytochemicals involved in their regulation (S3 Table). The network data provides a scope to explore the interrelationship of the target proteins with phytochemicals using computer-aided drug discovery approaches, which will be helpful in understanding the medicinal and multi-targeting potential of the herb in detail. Further, pleiotropic nature of the genes at the system level may also be investigated.

### Module and GO enrichment analysis of human PIN

**Fig 6:**
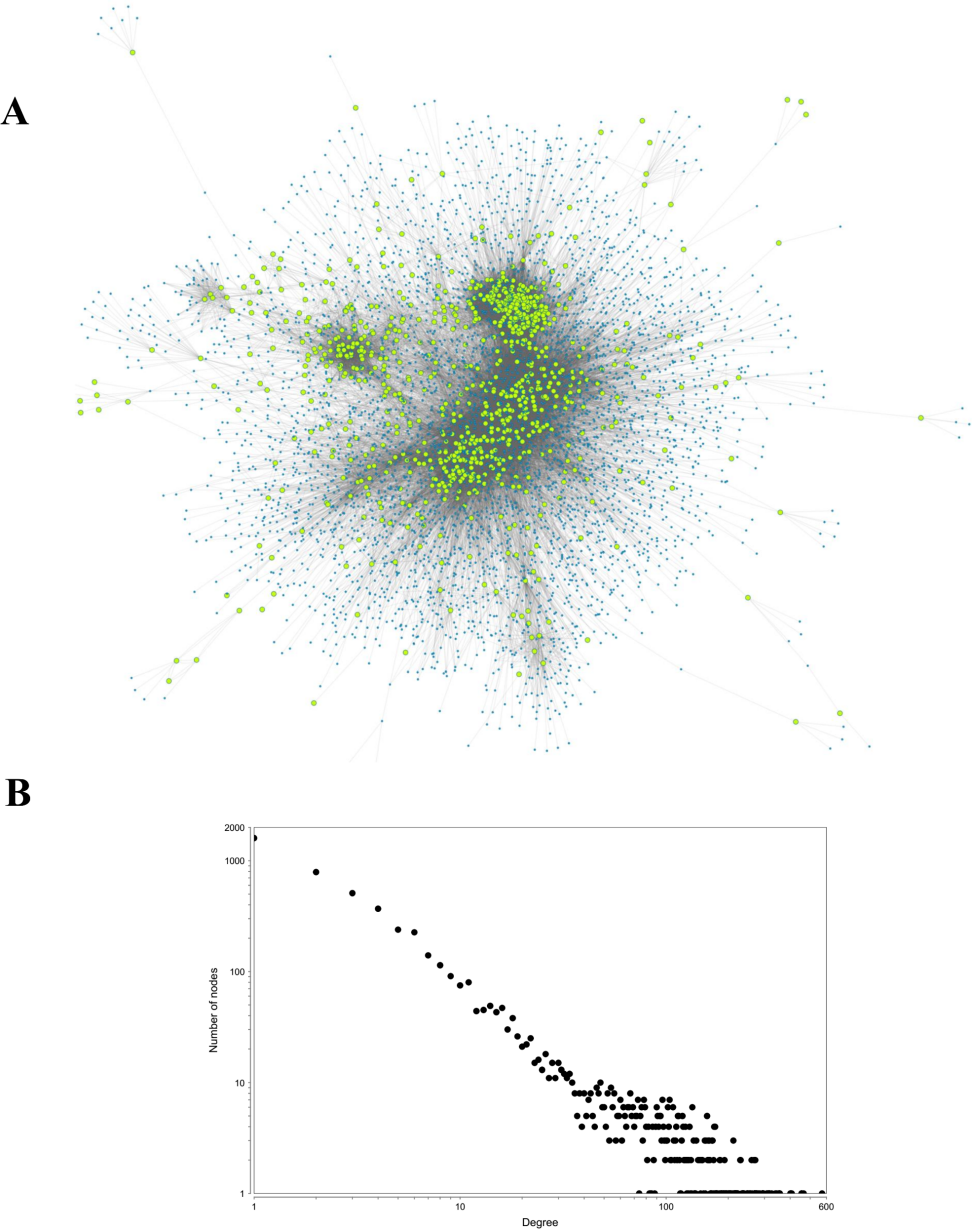
(A). Protein-protein interaction subnetwork of *Homo sapiens* targeted by phytochemicals of *P*. *longum*. First neighbours of all the targets proteins were mapped into the human PPI as obtained from STRING having high confidence level (score ≥ 900). Green highlighted nodes in the network represent the location of the target proteins of *P*. *longum*. (B) Node degree distribution of the PPI subnetwork. The neighbourhood connectivity of each node is represented using node degree distribution graph, analysed using Cytoscape. Both axes of the graph are represented in the logarithmic scale.

First-degree interactors of all the target proteins of *P*. *longum* were used to construct a subnetwork of the PIN of *Homo sapiens* (Fig 6). Topological analysis of the network shows that its degree distribution follows a power-law with *y* = 975.99*x*^-1.245^. The PIN was analysed using MCL clustering algorithm for module identification. Modules are shown in supplementary material (S1 Fig). Functional enrichment analysis of the modules with dense connection shows that *P*. *longum* exerts its effect mainly through regulating cell cycle, signal transduction, genetic information processing and metabolism machinery (Table 3).

**Table 3:** GO based biological processes of 15 highly connected modules in the protein-protein interaction subnetwork of *Homo sapiens* targeted by phytochemicals of *P*. *longum*.

Cluster-Number	GO-ID	P-value	Description
cluster-1	42770	1.61E-29	DNA damage response, signal transduction
cluster-2	23052	7.10E-28	Signaling
cluster-3	278	9.18E-122	Mitotic cell cycle
cluster-4	7186	2.09E-122	G-protein coupled receptor protein signaling pathway
cluster-5	6325	8.92E-63	Chromatin organization
cluster-6	7166	2.42E-47	Cell surface receptor linked signaling pathway
cluster-7	22900	2.05E-118	Electron transport chain
cluster-8	16055	1.77E-27	Wnt receptor signaling pathway
cluster-9	19220	1.48E-14	Regulation of phosphate metabolic process
cluster-10	6357	7.67E-21	Regulation of transcription from RNA polymerase II promoter
cluster-11	6096	8.75E-33	Glycolysis
cluster-12	6260	2.33E-48	DNA replication
cluster-13	9889	2.84E-14	Regulation of biosynthetic process
cluster-14	3735	1.28E-127	Structural constituent of ribosome
cluster-15	7049	1.44E-41	Cell cycle

Replication and repair mechanism (Module-1) involves proteins such as ATM, BLM and BRCA2. ATM is serine/threonine kinase gene and acts as an important cell-cycle checkpoint kinase [78]. BLM gene is a Bloom syndrome RecQ like helicase; protein encoded by this gene is involved in suppression of inappropriate recombination event in the cell [79]. BRCA gene helps in maintaining the stability of the genome. BRCA2 is involved in double-stranded DNA repair by regulating the homologous recombination pathway [80]. Mitotic cell cycle (Module-3) contains genes like ANAPC10, CDC 20 etc. ANAPC10 is a core subunit of anaphase-promoting complex and it is known that APC genes get altered in human colon cancer [81]. CDC20 (cell division cycle 20) acts as a regulatory protein and it has been shown *in vitro* that it is a promising therapeutic target for cancer treatment [82]. This shows that *P*. *longum* anti-cancerous activities are mainly due to the regulation of cell-cycle events and DNA-repair mechanisms.

It is commonly observed that disease occurrence is associated with the signal transduction failure, but the degree of its association varies greatly depending on the severity of disease. Wnt signaling pathways (Module-8) contain genes such as APC2, WNT7A and FRAT-1. Mutation in Wnt signaling pathway genes like APC (Adenomatous polyposis coli) is particularly evident in memory deficit cases. A gene knockout study in mice sample has shown that this gene plays an important role in the regulation of spinal locomotor activity and memory performance [83]. Its involvement in ocular, bone density disorders [84], [85] and colorectal cancer [86] are also well studied. Proteins of WNT gene family like WNT7A (Wnt family member 7A) encode secreted signaling proteins [87]. An earlier report in the developmental biology shows that Wnt7a is a highly conserved gene and plays an important role in early development of midbrain and telencephalon regions of the human brain [88]. Target data analysis shows that “P12644” encoded by BMP4 (Bone morphogenetic protein-4) is the target gene of WNT/beta-catenin signaling pathway [89]. Thus, it may be hypothesized that the herb’s nootropic effect is mainly associated with the proteins constituting this module.

*P*. *longum*’s anti-inflammatory quality may be linked to G-protein coupled signaling process (Module-4). This module contains genes like GRK2 and GRK6. Inflammatory mediators modulate GRKs signaling either by transcription regulation or its degradation. GRK2 act as a mediator in the pathway that causes inflammatory pain [90]. Thus, targeting GRKs by *P*. *longum* could be the reason of its anti-inflammatory properties. Pain relieving properties of this herb can be easily linked to the proteins in Module-4.

Proteins of module 10 and 14 are involved in genetic information processing by regulating transcription and translation machinery respectively. Genes like HES1, RUNX1, TGFB1, EP300 constitute module 10. HES1 is a hes family bHLH transcription factor-1. RUNX-1 is runt-related transcription factor 1 which participates in hematopoiesis. Its involvement in leukemia conditions is well documented [91]. TGFB1 is a transforming growth factor beta-1 and it regulates cell proliferation and differentiation, but shows an unregulated response in the tumor cells [92]. EP300 encodes E1-A associated cellular p300 transcriptional co-activator protein. It also helps in stimulating hypoxia-induce genes. A defect in the gene leads to Rubinstein-Taybi syndrome [93]. Thus, *P*. *longum* may be effective in improving transcriptional errors or the diseases associated with it.

Module 14 constitutes ribosomal proteins like RPL36, RPS3, RPL8, RPS7, RPS13 and RPL3. These proteins help in maintaining the structural integrity of the ribosomal assembly. The extra-ribosomal function of ribosomal proteins includes regulation of gene expression, cell-cycle control, regulation of apoptosis, modulation of DNA repair, regulation of development and differentiation, modulation of cell migration and invasion and regulation of angiogenesis [94]. Dysfunction of ribosome leads to a condition known as ribosomopathies. Although no data support the herb’s effectiveness in treating such dysfunction, but we believe that this area needs a detailed investigation. *P*. *longum* phytochemicals may have a regulatory effect on ribosomopathies.

Proteins of module-7 are present in the metabolic machinery which is reported to regulate Electron transport chain (ETC) and forms the basis of the energy production in the cell. These proteins are MT-CO1, UQCRC1 and NDUFA6. MT-CO1 (mitochondrial cytochrome c oxidase subunit-1), UQCRC1 (ubiquinol-cytochrome c reductase core protein) and NDUFA6 (NADH ubiquinone reductase subunit A6) are associated with the redox processes of ETC [95], [96], [97] and aid in an essential aspect of ATP generation. Abnormalities in the ETC chain are a characteristic feature of Alzheimer’s [98] and Parkinson’s [99] diseases of the brain. Thus, the chemical compounds produced in *P*. *longum* are involved in the crucial steps of ETC.

Glucose metabolism (Module-11) includes enzymes that are crucial for the conversion of glucose into pyruvate. This process is carried out by the cell to meet its energy requirements. The module contains gene like PGK2 (Phosphoglycerate kinase 2), ENO1 (Enolase 1), PKLR (Pyruvate kinase) and ALDOA (Aldolase, fructose-bisphosphate A). This shows that the herb may also be helpful in regulating energy metabolism. Additionally, high rate of glycolysis have been reported in cancerous cells [100]. In such condition, *P*. *longum*’s anti-cancerous property may be correlated with its interaction with the proteins of module involved in glucose metabolism.

### Drugability analysis and docking studies

**Fig 7:**
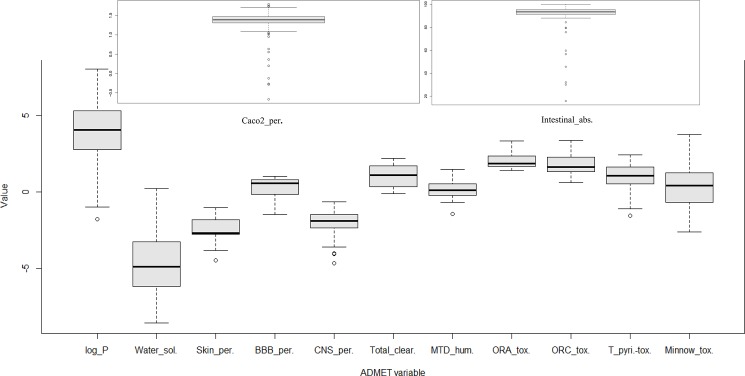
Box and whisker plots showing ADMET properties distribution of the *Piper longum’s* phytochemicals. The graph shows the plots of ADMET variables corresponding to octanal-water partition coefficient (log_P), water solubility (Water_sol.), skin permeability (Skin_perm.), blood-brain permeability (BBB_per), central nervous system permeability (CNS_perm.), total clearance (Total_clear.), maximum tolerated dose-humans (MTD_humans), oral rat acute toxicity (ORA_tox.), oral rat chronic toxicity (ORC_tox.), *T*. *Pyriformis* toxicity (T_pyri. tox), minnow toxicity (Minnow_tox), caco2 cell permeability (Caco2_per.) and intestinal absorption (Intestinal_abs.).

The estimation of ADMET and other drug-like properties are important to consider at an early stage of drug-discovery process, as the majority of drug candidates fail in clinical trials due to poor ADMET properties [101]. We evaluated the complete pharmacokinetic and toxicity profile of each phytochemical of *P*. *longum* for characterizing its drug likeliness (Fig 7) (S2 Table).

*In silico* estimation shows that the percentage intestinal absorption of the phytochemicals is more than 90% for 136 phytochemicals. 93% of the phytochemicals are likely to permeate the Caco2 cells as they have a permeability value greater than 1. Longumosides B (PL13), piperazine adipate (PL63), 2-amino-4-hydroxypteridine-6-carboxylic acid (PL67) and 4-[(1-Carboxy-2-methylbutyl) amino]-2(1H)-pyrimidinone (PL141) are predicted to have the least permeability among all other phytochemicals. The possible distribution of compounds through various compartments of the body was accessed using their blood-brain barrier (BBB) penetration and central nervous system (CNS) penetration coefficient. One of the criteria for a successful CNS drug is that the compound should not have binding affinity for P-glycoprotein [102]. The result shows that 111 phytochemicals may act as non-substrate of P-glycoprotein, thus possess the ability to be a potential CNS drug. Piperine (PL25) is known to have CNS acting power [103], the BBB and CNS penetration values were found to be -0.131 and -1.932 respectively. 115 phytochemicals of *P*. *longum* were showing the BBB and CNS penetration values similar to or greater than the piperine (PL25) values. This indicates that CNS targeting potential of other phytochemicals is noteworthy and should be explored further.

Lipinski’s “rule of five” criterion was adopted to estimate the likeliness of the phytochemicals to act as drug molecule [104]. 105 phytochemicals are shown to maintain the criteria of molecular weight less than 500 Dalton, number of hydrogen bond donors less than 5, hydrogen bond acceptors less than 10 and logP value (octanol-water partition coefficient) less than 5. Toxicity risk was also evaluated by checking Ames toxicity, oral-rat acute toxicity, oral-rat chronic toxicity, hepatotoxicity, cardiotoxicity, *T*. *pyriformis* toxicity and Minnow toxicity. 13% of the compounds are predicted to be positive for hepatotoxicity. The cardiotoxicity was evaluated with hERG (human ether-a-go-go-related gene) inhibition. Interestingly, not a single phytochemical is positive for hERG1 inhibition, which reflects the cardioprotective nature of *P*. *longum*.

The final screening of all the parameters in phytochemical dataset resulted in identification of 20 phytochemicals that possess a high probability of acting as effective lead molecules (S2 Table). For identifying the novel regulatory molecules to the previously discussed 5 key protein targets, these 20 phytochemicals were back-mapped in the PC-PT network. This resulted in the selection of 7 phytochemicals that were forming 16 phytochemical-protein target pairs. To estimate the binding affinity of these potential phytochemical-protein target pairs, docking studies were performed. This is essential to identify the best fit between the phytochemical and protein molecule, both in terms of energy and geometry. Binding energy calculations of each pair is represented in Table 4. Chemical features of all these phytochemicals can be explored further to design their synthetic analogs with optimised pharmacological activity.

**Table 4:** Docking energies of potential protein targets with their corresponding regulatory phytochemicals.

Protein Targets (Uniprot ID)	PDB ID	Phytochemical ID	Docking energy (Kcal/mol)
P04150	4MDD	PL125	-4.72
P21397	2BXR	PL6	-8.94
		PL61	-4.68
		PL81	-4.49
		PL95	-4.39
		PL125	-8.47
		PL152	-7.4
P27338	2BXS	PL6	-8.71
		PL61	-4.31
		PL81	-4.84
		PL95	-4.63
		PL125	-7.7
		PL152	-7.19
P37231	4EMA	PL125	-7.6
Q8NER1	3J5P	PL6	-9.04
		PL71	-4.59

### A case-study on the *Piper longum*’s action on neurological diseases and disorders

To further explore the neuromodulatory prospectives of the *P*. *longum*, curated gene-disease associations corresponding to nervous system diseases and mental disorders were selected from PC-PT-DA network. This resulted in the identification of 384 protein targets, out of these 215 were FDA-approved protein targets as listed in Drugbank. These proteins were back-mapped to PC-PT-BP network for selecting their interactions with previously shortlisted 20 potential drug-like phytochemicals.

In this way, we could derive a sub-network specific to neurological diseases and consisting of druggable phytochemical—protein target pairs from the PC-PT-BP network of *P*. *longum* (S2 Table). In this sub-network, it is observed that metabolic pathways (path:hsa01100) and neuroactive ligand-receptor interaction (path:hsa04080) were highly enriched, implying that multiple proteins from the dataset exert their biological functions mainly through these pathways. This result is in confirmation with the earlier findings showing the atypical role of impaired metabolic pathway processes in various neurological diseases and disorders like Alzheimer etc. [105].

**Fig 8:**
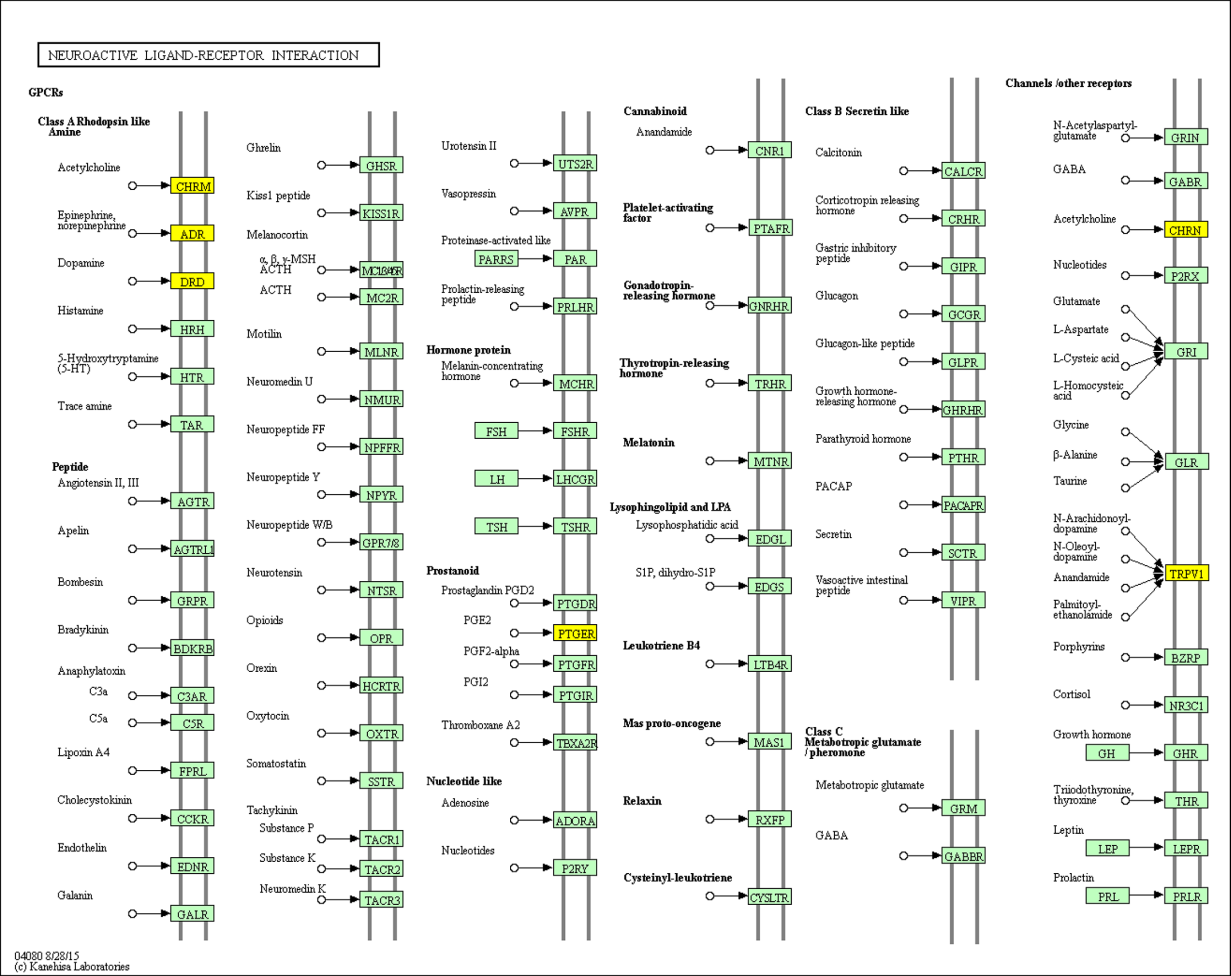
Neuroactive ligand receptor interaction pathway (path:hsa04080) obtained from KEGG database. The mapped genes corresponding to CHRM, ADR, DRD, PTGER, CHRN and TRPV1 are marked in yellow.

Identification and inquiry of neuroactive pathways are crucial for the design and development of improved therapeutic strategies against nervous system disorders like Schizophrenia [106] and Parkinson’s disease [107]. Therefore, the derived sub-network is analyzed in detail with specific focus to highlight the position and role of protein targets in the neuroactive ligand-receptor interaction pathway. We could map 11 proteins from 6 gene classes to this pathway and are shown as yellow rectangles in Fig 8. To figure out if these proteins participate in a specific biological process, these were back mapped to the module classes of the human PIN (detailed in section corresponding to module and GO enrichment analysis). 6 proteins out of 11 were showing their involvement in “G-protein coupled receptor protein signaling pathway” corresponding to Module 4 (S2 Fig). This illustrates that these proteins (corresponding to the gene class CHRM, ADRA, DRD, PTGER1) mutually contribute to the G-protein coupled signaling process. The alteration of the signaling process, especially dysfunction of GPCRs, has been a cause for the pathological changes within a brain region. In recent years, targeting potential of GPCR heteroreceptor complexes specific to CNS region is being explored to provide new insights towards the field of neuropharmacology. They have become impressive targets for neurological and mental disorders like schizophrenia, anxiety, depression and Parkinson’s disease [108].

**Fig 9:**
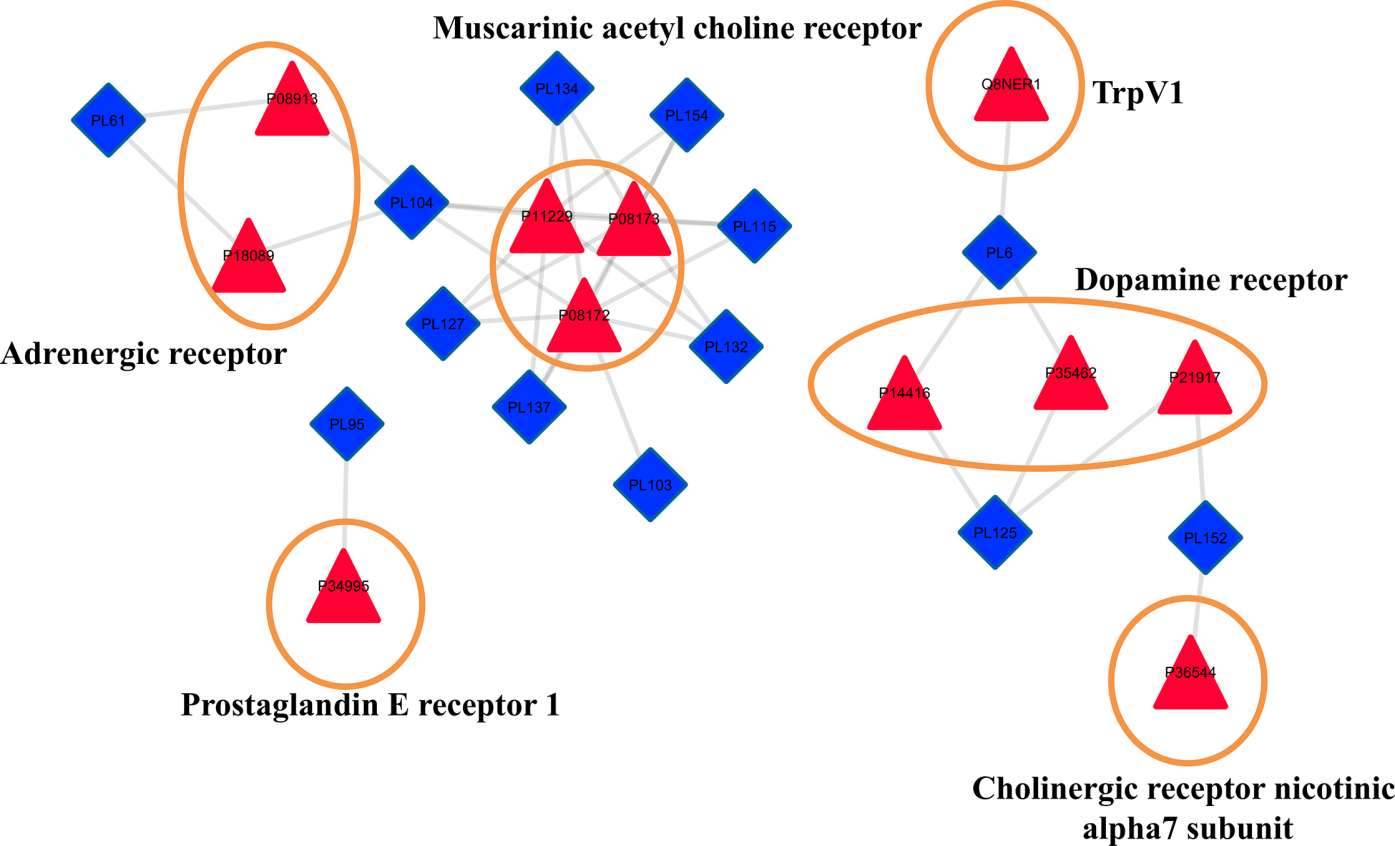
Potential DPC-PTN network. DPC (blue rhombus) stands for Drugabble PhytoChemicals from *P*. *longum* and PTN (red triangle) stands for Protein Targets selected from Neuroactive-ligand interaction pathway (path:hsa04080). 11 potential protein targets (covering 6 gene classes) for neurological disease and disorders from “path:hsa04080” are regulated by 14 potential drug-like phytochemicals of *P*. *longum*. Edge of the network represents the possible regulatory phytochemical partner for each protein target. Proteins are grouped according to their gene class. Three gene classes corresponding to adrenergic receptor (ADA), Muscarinic acetyl choline receptor (CHRM) and Dopamine receptor (DRD) constitute multiple proteins, while remaining three corresponding to Transient receptor potential cation channel subfamily V member 1 (TrpV1), Prostaglandin E receptor 1 (PTGER1) and Cholinergic receptor nicotinic alpha7 subunit (CHRNA7) are specific for a particular protein.

Phytochemical mapping of the 11 proteins highlights the role of 14 potential drug-like phytochemicals in their regulation. It was observed that sesamol (PL95) has a one-to-one regulatory relationship with PTGER1 (P34995) in this pathway, while three phytochemicals (PL6, PL104 and PL152) have multi-targeting potential for regulating more than one protein from the rest of 5 gene classes (Fig 9).

PTGER1 is a prostaglandin E receptor 1 (EP1), one of the four G-protein coupled receptors for PGE2 (Prostaglandin E2) that are EP1, EP2, EP3 and EP4. PGE2 is the most abundant eicosanoid in the human system that shows a range of paracrine and autocrine effects by binding to these GPCRs [109], [110], [111] and PGE2 pathway is also known to be acting as a novel biomarker against antipsychotic treatment [106]. The possible molecular interaction between PL95 and PTGER1 was studied using Autodock Vina that estimated the binding affinity to be -4 kcal/mol. As only few inhibitors are available for the PGE2 receptors, the inhibitory activity of PL95 can be explored further for developing novel chemoprotective and antipsychotic agents.

Multi-targeting phytochemical screening identifies PL6, PL152 and PL104 as a common ligand for two different receptors. Selection of such phytochemical that targets multiple genes, adds an extra advantage towards their choice as a drug candidate. To evaluate the molecular interactions of these phytochemicals and their interacting protein partners from the given pathway, complexes of enzymes and ligands were obtained using Autodock Vina. Binding affinity calculation for each pair is shown in Table 5.

**Table 5:** Molecular docking results of multi-targeting phytochemicals involved in nervous system diseases and disorders.

	Muscarinic Acetyl choline receptor	Cholinergic receptor nicotinic alpha7 subunit	Adrenergic receptor	Dopamine receptor	Transient receptor potential cation channel subfamily V member 1
PL152		P36544 (-5.63 kcal/mol)		P21917 (-6.03 kcal/mol)	
PL6				P14416 (-6.57 kcal/mol)	Q8NER1 (-5.72 kcal/mol)
				P35462 (-7.99 kcal/mol)	
PL104	P11229 (-5.66 kcal/mol)		P08913 (-5.34 kcal/mol)		
	P08172 (-5.29 kcal/mol)		P18089 (-3.70 kcal/mol)		
	P08173 (-5.24 kcal/mol)				

Binding energy value for each pair is represented in parentheses.

**Fig 10:**
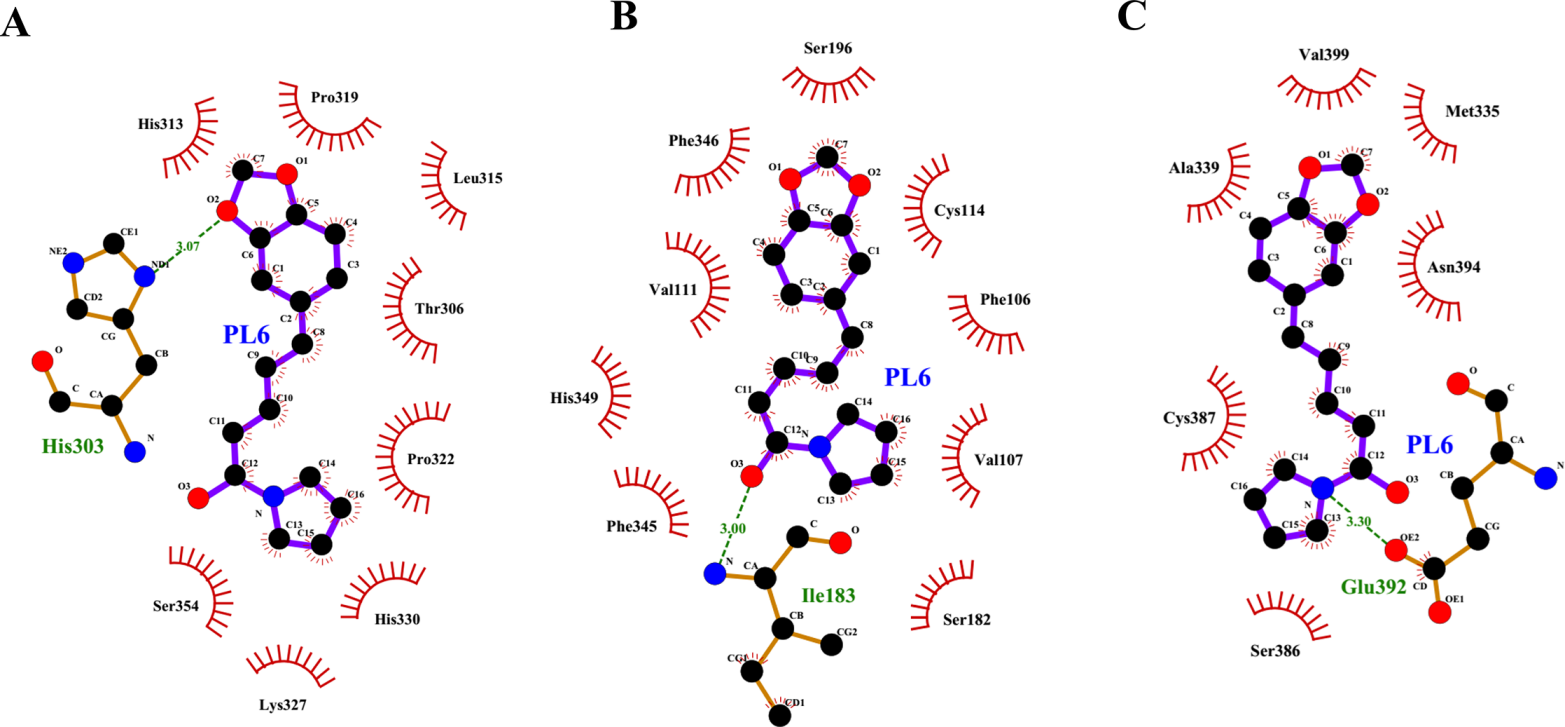
Hydrophobic interactions and hydrogen bonding between PL6 and its targets. **Two dimensional representation of interaction observed between PL6 and its interacting proteins listed in Table 5. (A)** Ligplot^+^ analysis of the docked complex of PL6 and P14416: Dopamine receptor DRD2. **(B)** Ligplot^+^ analysis of the docked complex of PL6 and P53462: Dopamine receptor DRD3. **(C)** Ligplot^+^ analysis of the docked complex of PL6 and Q8NER1 (TrpV1). Protein residues involved in hydrophobic interactions are represented as arcs and hydrogen bonding with dashed lines.

Molecular interactions of PL6 that is a potential regulator of protein targets from two gene classes, DRD (Dopamine receptor) and TrpV1 (Transient receptor potential cation channel subfamily V member 1), are studied in detail. Altered functioning of the DRD2 receptor (corresponding to P14416 in our dataset) has been a cause for schizophrenia and search of novel antipsychotics corresponding to DRD2 receptor antagonists are still in process [98]. Similarly, TrpV1 (corresponding to Q8NER1) present in the brain is a key protein for microglia and neuron communication. Neurobehavioral studies show their involvement in neurological and psychiatric disorders like epilepsy, depression etc. [111]. There has been a continuous search of the antagonists for TRPV1 as anti-inflammatory agents like capsaicin and resiniferatoxin (RTX) for the treatment of neuropathic pain [112]. Ligplot^+^ analysis of the PL6 and Q8NER1 complex highlights the role of Glutamic acid (Glu:392) in the interaction. Similarly, the analysis of docking complex of PL6 and its interacting protein corresponding to other class of membrane receptors (P14416: Dopamine receptor: DRD2) shows that the compound is able to form hydrophobic interactions with the protein molecule. Further, Histidine residue (His:303) of the protein is involved in the formation of a hydrogen bond with PL6 while for P35462 (Dopamine receptor: DRD3) Isoleucine (Ile:183) is hydrogen bonded (Fig 10).

Using the similar strategy, crucial pathways associated with other disease classes can be analyzed and explored for the detailed information about their regulatory phytochemicals. We hope that methodology developed in this work will open a new way to explore the drug-like molecules from natural herbs for disease management.

## Summary

The traditional Indian medicine (TIM) system commonly known as Ayurveda is a more than four thousand years old heritage of the Indian subcontinent and is a huge repository of information about multiple natural medicines for their therapeutic potential. *P*. *longum* is an important constituent of many Ayurvedic formulations and is most widely used as a part of “Trikatu”. However, the multi-targeting potential of this herb and underlying mechanism of its cellular-level action are still unexplored. In the current study, we have examined the medicinal effects of *P*. *longum* using network pharmacology, an approach that has emerged in recent years as a key route to investigate the healing potential of the traditional herbs for drug discovery and drug development procedures. Our methodology involved literature survey, database mining, drug-likeliness prediction, phytochemical-protein target, and protein target-disease relationship study to examine the multifaceted potential of this herb. Among 159 phytochemicals, 20 are estimated to be the potential lead molecules based on the drug-likeliness filter. 14 of these phytochemicals were found to be regulators of protein targets involved in the nervous system related diseases and disorders. These phytochemicals affect the signaling process of the neuroactive ligand interaction pathway *via* regulation of 11 proteins. Specific and multi-targeting potential of 4 of these compounds were further explored via molecular docking studies for their possible usage in neuro pharmacotherapy. We expect that this work based on systems-level network assisted studies of *P*. *longum* will offer a new way to look upon the hidden potentials of this herb. The data obtained from docking analysis may be taken for *in vitro* studies, which may eventually be helpful in identification of novel and effective lead molecules from a natural pool of compounds present in *P*. *longum*. We are hopeful that this study will prove to be an important basis for understanding the phytochemical-protein level coordination in various Ayurvedic formulae that use *P*. *longum* as an integral part. The phytochemical-protein target and disease relationship represented in the form of interaction networks will be helpful in understanding the underlying molecular mechanism in detail. We believe that the comprehensive computational approach developed in this work involving multi-level studies of phytochemical-protein target interaction identification will be helpful to screen natural lead like compounds against various diseases.

## Supporting information

S1 Fig Modules identified in the subnetwork of the PIN of *Homo sapiens*, constructed using the first-degree protein interactors of all the target proteins of *P*. *longum*. The modules were identified using MCL (Markov Cluster) algorithm.
(TIF)

S2 Fig Mapping of the proteins associated with neuroactive–ligand interaction pathway in the module 4 corresponding to “G-protein coupled receptor protein signaling pathway”. (TIF)

S1 Table Information of the 1109 protein targets of *P*. *longum* with their KEGG pathway mapping. (XLSX)

S2 Table ADMET values of the phytochemicals of *P*. *longum* (including the listing of 20 putative lead phytochemicals). (XLSX)

S3 Table List of potential protein targets (identified by 3 of the 4 protein target prediction softwares) with their interacting phytochemical information. (XLSX)

## Abbreviations

ADMET: Absorption, Distribution, Metabolism, Excretion and Toxicity
BP: Biological pathways
DA: Disease association
GPCR: G protein-coupled receptors
*P*. *longum*: *Piper* *longum*
PC: Phytochemical
PCt: Number of protein targets corresponding to a particular phytochemical
PPI: Protein protein interactions
PT: Protein target
Tt: Total number of protein targets of *P*. *longum*
