# Supplementary figures and images for: A census of P. longum’s phytochemicals and their network pharmacological evaluation for identifying novel drug-like molecules against various diseases, with a special focus on neurological disorders
Source: PLoS One. 2018 Jan 10;13(1):e0191006. doi: 10.1371/journal.pone.0191006 (PMC5761900; doi:10.1371/journal.pone.0191006)

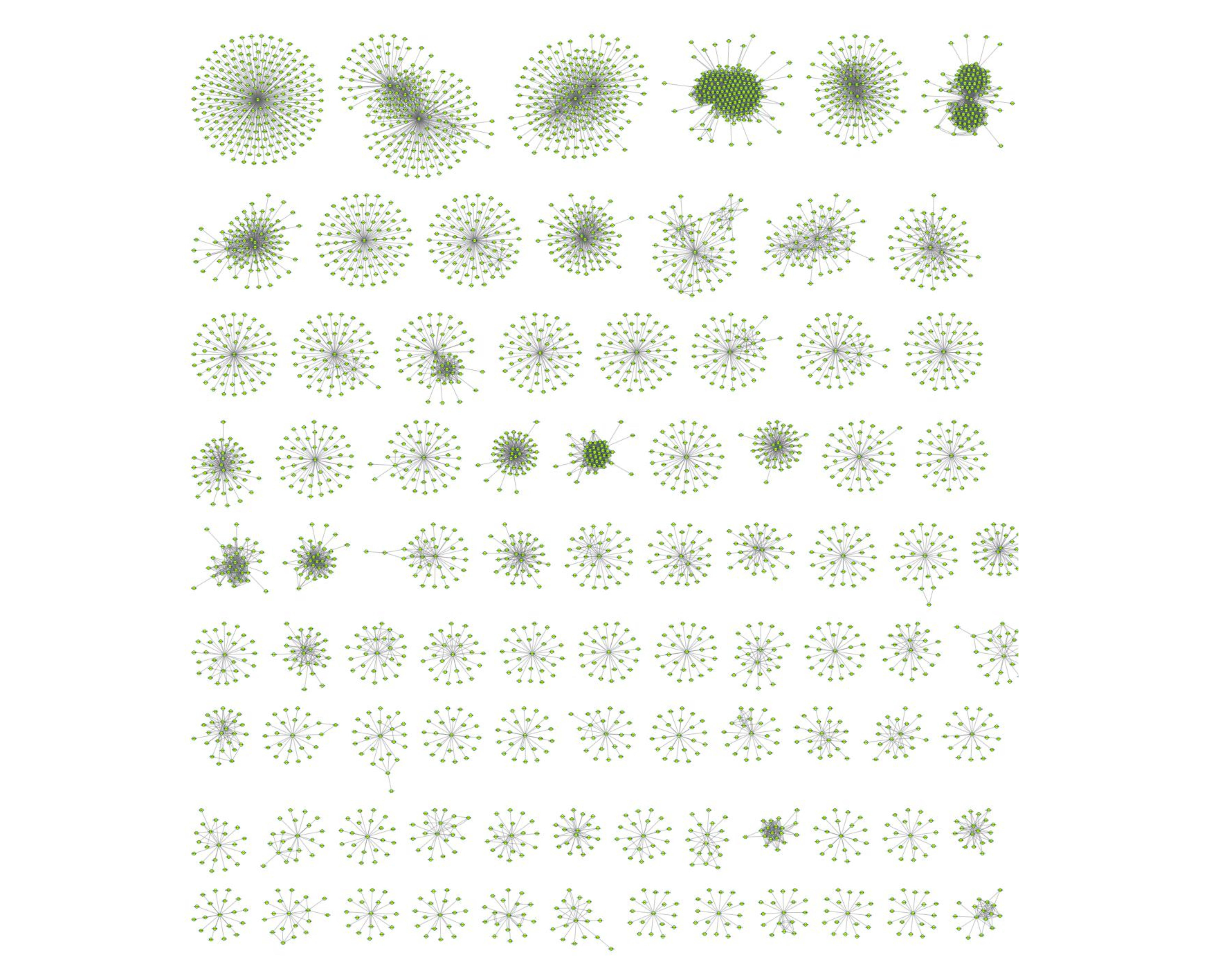

Supplement: S1 Fig — The modules were identified using MCL (Markov Cluster) algorithm. (TIF) [file pone.0191006.s001.tif]

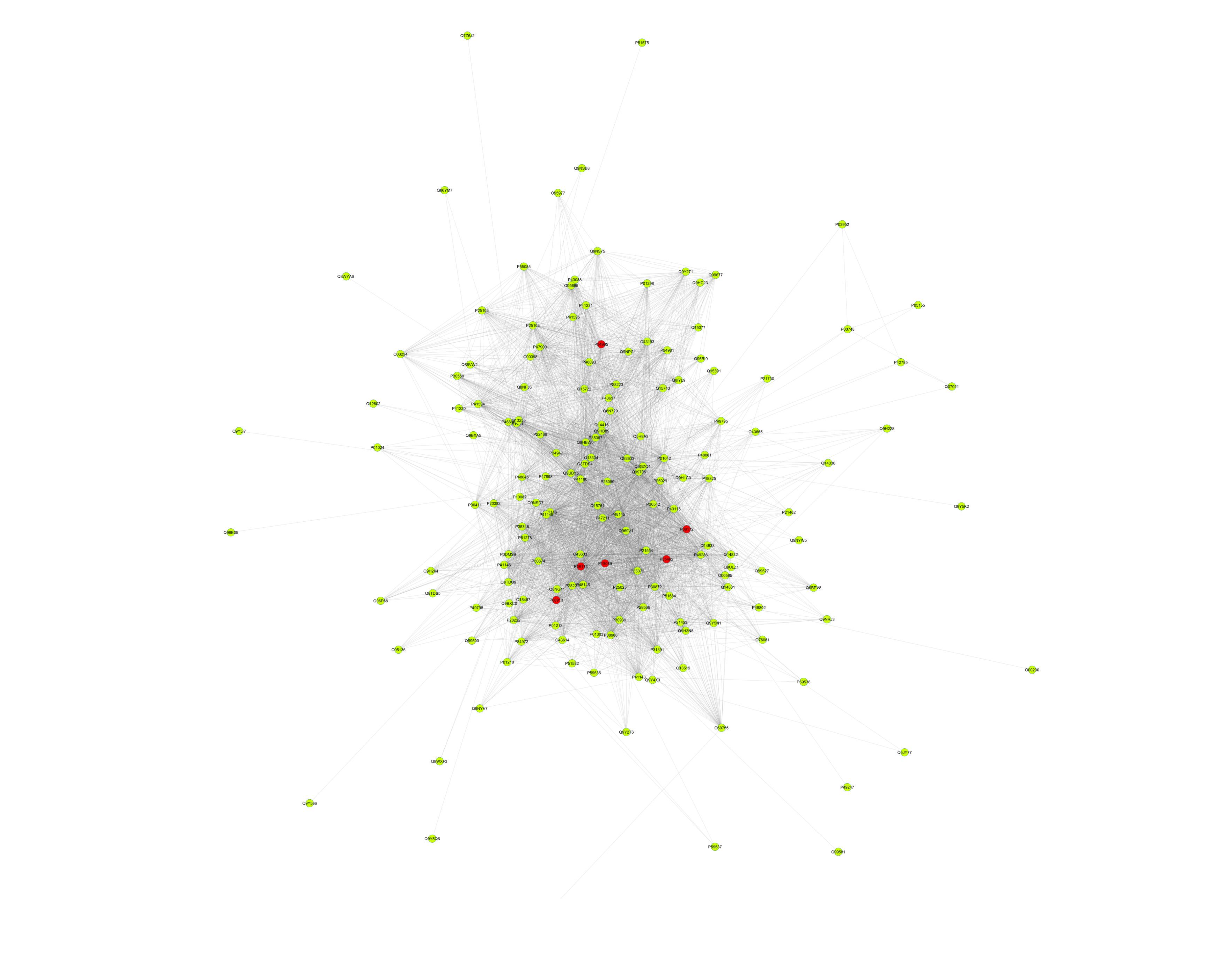

Supplement: S2 Fig — (TIF) [file pone.0191006.s002.tif]
